# Latent circuit inference from heterogeneous neural responses during cognitive tasks

**DOI:** 10.1038/s41593-025-01869-7

**Published:** 2025-02-10

**Authors:** Christopher Langdon, Tatiana A. Engel

**Affiliations:** 1 Princeton Neuroscience Institute, Princeton University, Princeton, NJ USA; 2 Cold Spring Harbor Laboratory, Cold Spring Harbor, NY USA

**Keywords:** Neural circuits, Network models, Dynamical systems, Decision, Cognitive control

## Abstract

Higher cortical areas carry a wide range of sensory, cognitive and motor signals mixed in heterogeneous responses of single neurons tuned to multiple task variables. Dimensionality reduction methods that rely on correlations between neural activity and task variables leave unknown how heterogeneous responses arise from connectivity to drive behavior. We develop the latent circuit model, a dimensionality reduction approach in which task variables interact via low-dimensional recurrent connectivity to produce behavioral output. We apply the latent circuit inference to recurrent neural networks trained to perform a context-dependent decision-making task and find a suppression mechanism in which contextual representations inhibit irrelevant sensory responses. We validate this mechanism by confirming the behavioral effects of patterned connectivity perturbations predicted by the latent circuit model. We find similar suppression of irrelevant sensory responses in the prefrontal cortex of monkeys performing the same task. We show that incorporating causal interactions among task variables is critical for identifying behaviorally relevant computations from neural response data.

## Abstract (web summary)

The latent circuit model identifies low-dimensional mechanisms of task execution from heterogenous neural responses. This approach reveals a latent inhibitory mechanism for context-dependent decisions in neural network models and the prefrontal cortex.

## Main

Cognitive functions depend on higher cortical areas, which integrate diverse sensory and contextual signals to produce a coherent behavioral response. These computations result from interactions between excitatory and inhibitory neurons in cortical circuits. Traditionally, hand-crafted neural circuit models were used to pose specific mechanistic hypotheses about how excitation and inhibition between a few neural populations representing task variables control the flow of information from input to behavioral output^1–11^. Because these circuit models usually assume a relatively simple connectivity structure, their connectivity can be directly related to a dynamical system description of computations supporting cognitive task execution^5,6^. Thus, these models explicitly specify a circuit mechanism in the connectivity structure that gives rise to a dynamical mechanism controlling the flow of neural trajectories to implement task computations. By linking connectivity to neural dynamics and behavioral output, these models can predict changes in dynamics and behavioral performance under perturbations of the circuit structure (for example, changes in excitation–inhibition balance^12^) and thus can be causally validated in experiments^13–15^. However, hand-crafted circuit models come short of capturing the complexity and heterogeneity of single-neuron responses in the cortex.

Single neurons in areas such as the prefrontal cortex (PFC) show complex heterogeneous tuning to multiple task variables^16–21^, which presents a formidable challenge for identifying underlying circuit mechanisms. Similar heterogeneous responses emerge in high-dimensional recurrent neural network (RNN) models trained to perform cognitive tasks^19,22–24^ or reproduce neural activity data^25–29^ by optimizing recurrent connectivity parameters. Although trained RNNs are a class of neural circuit models, the complexity of their high-dimensional activity and connectivity obscures the interpretation of circuit mechanisms in these networks^30^. It has been possible to determine dynamical mechanisms of task computations by either characterizing fixed points and linearized dynamics around them in RNNs^19,31^ or fitting a dynamical system directly to neural response data^27,32–35^. These approaches, however, provide no insight into how particular features of the flow field in a dynamical system arise from the network connectivity. Thus, high-dimensional RNNs currently serve as intermediate mechanistic models of heterogeneous neural responses, which yield dynamical mechanisms but leave the underlying circuit mechanisms unknown.

**Fig. 1:**
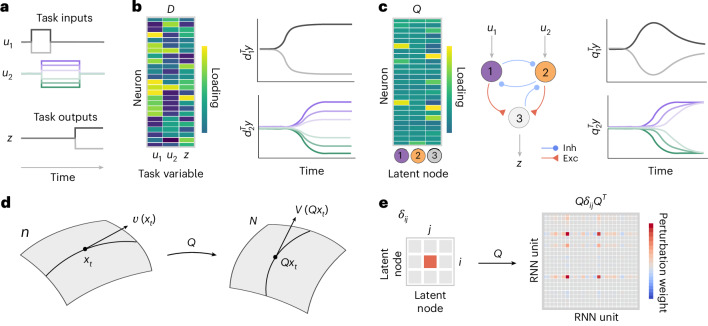
Latent circuit model of heterogeneous neural responses during cognitive tasks. **a**, A cognitive task requires the production of desired behavioral outputs *z* prompted by external inputs *u*. The inputs *u* and outputs *z* are the task variables. **b**, Dimensionality reduction based on the correlation between neural activity and task variables. The matrix *D*^*T*^ defines a projection from neural activity space onto task variables (left). Each column of *D* defines an axis in the neural population state space such that the projection of neural activity onto this axis correlates with a specific task variable (right). **c**, Latent circuit model. Embedding matrix *Q* defines a projection from the population state space onto nodes of the latent circuit (left). The nodes interact through recurrent dynamics Eq. (2), are driven by task inputs *u* and generate task outputs *z* (center). Each column of *Q* defines an axis in the population state space such that the projection of neural activity onto this axis correlates with the activity of one node in the latent circuit (right); Inh, inhibitory; Exc, excitatory. **d**, We differentiate the mapping of trajectories *y* = *Qx* to obtain the correspondence between the vector field $\dot{y}=V(y)$ of the high-dimensional system and the vector field $\dot{x}=v(x)$ of the latent circuit: *V*(*y*) = *Qv*(*x*). This equation states that the subspace spanned by the columns of *Q* is an invariant subspace of the high-dimensional system; that is, the vector field at any point in this subspace lies entirely in this subspace. Using the orthonormality of *Q*, we then derive the relationship: *Q*^*T*^*V*(*Qx*) = *v*(*x*), which asserts that the latent vector field *v*(*x*) describes dynamics of the high-dimensional system in this invariant subspace. **e**, The relationship between connectivity of the latent circuit and RNN enables us to translate connectivity perturbations. Perturbing connection *δ*_*ij*_ from node *j* to node *i* in the latent circuit maps onto rank-one connectivity perturbation $Q{\delta }_{ij}{Q}^{T}={q}_{i}{q}_{j}^{T}$ in the RNN.

Although single-neuron responses are complex and heterogeneous, their joint population activity is often low dimensional across many cognitive tasks and brain areas^30,36^. Accordingly, dimensionality reduction methods are commonly used to reveal representations of low-dimensional latent variables in neural population activity, which reflect computations emerging at the population level. Because unsupervised methods do not explicitly model task inputs and behavioral outputs, the latent variables they infer may be unrelated to the cognitive task execution^26,37–40^. Therefore, targeted dimensionality reduction methods directly model neural representations of task inputs and behavioral outputs by seeking low-dimensional projections of neural population activity that correlate with external task variables^19,21,41–43^ (Fig. 1a,b). However, unlike RNNs and circuit models, these correlation-based methods do not incorporate recurrent interactions among task variables, which implement computations necessary to solve the task. Hence, it remains uncertain whether neural representations uncovered by these methods bear any relevance for driving behavior and have the causal predictive power comparable to the dynamical and circuit mechanisms.

The glaring gap between circuit mechanisms and correlation-based dimensionality reduction methods is apparent in studies of the PFC’s role in context-dependent decision-making. Context-dependent decision-making requires flexible trial-by-trial switching between alternative stimulus–response mappings. Most circuit models hypothesize a relatively simple mechanism based on inhibition of sensory representations irrelevant in the current context^8–10,44–46^. By contrast, dimensionality reduction methods applied to PFC data or RNN activity show minimal suppression of irrelevant sensory responses^19,21^, seemingly invalidating the inhibitory circuit mechanism. These results suggest that RNNs and conceivably the PFC implement qualitatively novel task solutions that do not exist in small circuits and emerge only in high-dimensional networks^19,47,48^. However, because correlation-based dimensionality reduction methods bear no links to the connectivity and causal mechanisms, whether heterogeneous neural responses during cognitive tasks arise from low-dimensional circuit mechanisms remains an open question.

To bridge this gap, we develop the latent circuit model, a dimensionality reduction approach that jointly fits neural responses and task behavior and incorporates recurrent interactions among task variables to capture a causal mechanism of task execution. Our model fits neural responses with dynamics generated by a low-dimensional latent circuit, thereby directly testing the hypothesis that heterogeneous neural responses arise from a low-dimensional circuit mechanism. The model simultaneously infers a low-dimensional latent circuit connectivity generating task-relevant dynamics and heterogeneous mixing of these dynamics in single-neuron responses. We applied latent circuit inference to RNNs optimized on a context-dependent decision-making task and found a circuit mechanism based on the inhibition of irrelevant sensory representations. We validated this mechanism by confirming the behavioral effects of patterned perturbations of the RNN activity and connectivity predicted by the latent circuit model. Moreover, fitting the latent circuit model to neural recordings from the PFC of monkeys performing the same context-dependent decision-making task revealed a qualitatively similar suppression mechanism, in contrast to previous analyses of the same data with correlation-based methods^19,21^. Using RNN perturbations, we show that dimensionality reduction methods that do not incorporate causal interactions among latent variables are biased toward uncovering behaviorally irrelevant representations. Our results show that high-dimensional networks use low-dimensional circuit mechanisms, establish the feasibility of inferring these mechanisms from neural response data and open new possibilities of causally validating circuit mechanisms in perturbation experiments.

## Results

### Latent circuit model

To bridge the gap between dimensionality reduction, circuit mechanisms and single-neuron heterogeneity, we develop a latent circuit model (Fig. 1c). Similar to other dimensionality reduction methods, we model high-dimensional neural responses $y\in {{\mathbb{R}}}^{N}$ (*N* is the number of neurons) during a cognitive task using low-dimensional latent variables $x\in {{\mathbb{R}}}^{n}$ as

1 $$y=Qx,$$

where $Q\in {{\mathbb{R}}}^{N\times n}$ is an orthonormal embedding matrix and *n* ≪ *N*. The latent variables *x* are constrained to be nodes in a neural circuit with dynamics

2 $$\dot{x}=-x+f({w}_{{\textrm{rec}}}x+{w}_{{\textrm{in}}}u),$$

where *f* is a rectified linear (ReLU) activation function. The latent nodes interact via the recurrent connectivity *w*_rec_ and receive external task inputs *u* through the input connectivity *w*_in_. We also require the latent circuit to perform the task, that is, we can read out the task outputs *z* from circuit activity via the output connectivity *w*_out_:

3 $$z={w}_{{\rm{out}}}x.$$

The latent circuit model captures task-related neural activity in the low-dimensional subspace spanned by the columns of *Q*, with dynamics within this subspace generated by the neural circuit Eq. (2). We infer the latent circuit parameters (*Q*, *w*_rec_, *w*_in_ and *w*_out_) from neural activity *y* by minimizing the loss function *L* = ∑_*k*,*t*_∥*y* − *Qx*∥_2_ + ∥*z* − *w*_out_*x*∥_2_, where *k* and *t* index trials and time within a trial, respectively (Methods).

In the latent circuit model, the heterogeneity of single-neuron responses has three possible sources: mixing of task inputs to the latent circuit via *w*_in_, recurrent interactions among latent nodes via *w*_rec_ and linear mixing of representations in single neurons via the embedding *Q*. The orthonormality constraint on *Q* implies that the projection defined by the transpose matrix *Q*^*T*^ is a dimensionality reduction in which projection onto the *i*th column of *Q* correlates with the activity of the *i*th node in the latent circuit. Conversely, the image of each latent node *i* is a high-dimensional activity pattern given by the column *q*_*i*_ of the matrix *Q*. Thus, the latent circuit provides a dimensionality reduction that incorporates an explicit mechanistic hypothesis for how the resulting low-dimensional dynamics are generated.

In general, it is not obvious under what circumstances we can satisfactorily fit a latent circuit model to the responses of a high-dimensional system. If, for example, solutions to cognitive tasks that emerge in large systems are qualitatively different from mechanisms operating in small circuits, then we should not be able to adequately fit task-related dynamics of the large system with a low-dimensional circuit model. However, the existence of a low-dimensional circuit solution that accurately captures dynamics of the large system would suggest that this circuit mechanism may be latent in the high-dimensional system.

### Interpreting latent connectivity

The advantage of the mechanistic model for latent dynamics is that we can interpret the latent connectivity and relate it to the connectivity of the high-dimensional system. In this context, RNNs optimized to perform a cognitive task provide an ideal setting for testing and validating the latent circuit inference. RNNs mimic the heterogeneity and mixed selectivity of neural responses in the cortex during cognitive tasks while providing full access to each unit’s activity, network connectivity and behavioral outcomes.

To interpret the latent connectivity, we differentiate the embedding Eq. (1) to obtain the correspondence between vector fields of the high-dimensional and low-dimensional dynamical systems (Fig. 1d and Methods). We can then derive an explicit relationship between connectivity matrices of a high-dimensional RNN and a low-dimensional latent circuit. We consider RNNs with dynamics

4 $$\dot{y}=-y+f({W}_{{\rm{rec}}}\,y+{W}_{{\rm{in}}}u),$$

where *W*_rec_ and *W*_in_ are the recurrent and input connectivity matrices, respectively (Methods). Using the fact that the vector fields of the RNN and latent circuit are piecewise-linear dynamical systems, we derive a relationship between their recurrent and input connectivity matrices (Methods):

5 $${Q}^{T}{W}_{{\rm{rec}}}Q={w}_{{\rm{rec}}},\;{Q}^{T}{W}_{{\rm{in}}}={w}_{{\rm{in}}}.$$

The relation Eq. (5) shows that the latent circuit connectivity *w*_rec_ is a low-rank structure in the connectivity of a high-dimensional network, which captures interactions among the latent variables defined by the columns of *Q*. This relation does not necessarily imply that the full recurrent connectivity *W*_rec_ is low rank^47,49,50^. Rather, it is a weaker condition that the linear subspace defined by *Q* is an invariant subspace of the high-dimensional recurrent connectivity matrix. Because, in practice, we search for the latent circuit by minimizing the loss function *L*, if *L* is not exactly equal to 0, then Eq. (5) holds only approximately.

The relation between connectivity matrices has the powerful consequence that we can validate the latent circuit mechanism directly in the RNN connectivity. First, if the latent circuit faithfully describes the mechanism operating in the RNN, by conjugating the RNN connectivity matrix with *Q* (Eq. (5)), we expect to find low-dimensional connectivity structure similar to the latent circuit connectivity. Such an agreement is nontrivial because the latent circuit inference uses only RNN activity without knowledge of the RNN connectivity. Second, Eq. (5) enables us to translate connectivity perturbations in the latent circuit onto the connectivity perturbations in the RNN. Specifically, a change in the connection *δ*_*ij*_ between nodes *i* and *j* in the latent circuit maps onto a rank-one perturbation of the RNN connectivity matrix (Fig. 1e and Methods),

6 $${\delta }_{ij}\to {q}_{i}{q}_{j}^{T},$$

where *q*_*i*_ is the *i*th column of *Q*. By translating latent connectivity perturbations onto the RNN, we can verify whether these connectivity perturbations affect RNN behavioral performance as predicted by the latent circuit model. The validation of inferred circuit mechanisms via RNN perturbations is critical because the fit quality alone does not guarantee that the inferred model captures the correct mechanism that generated data^40^. Thus, confirming predicted behavioral effects of connectivity perturbations establishes the existence of the inferred low-dimensional circuit mechanism in the RNN.

### Latent circuit for context-dependent decision-making

**Fig. 2:**
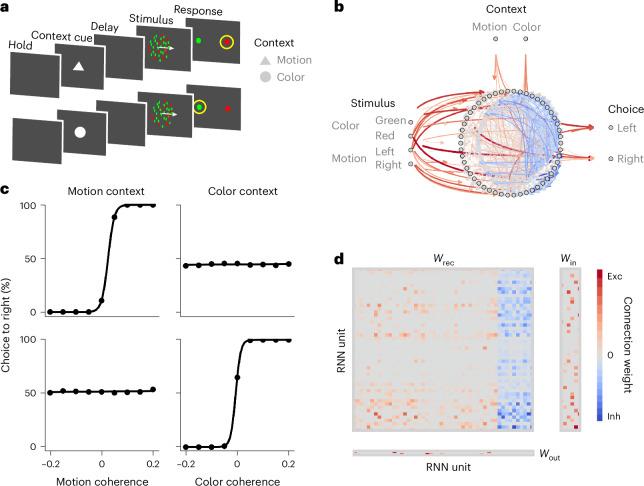
RNN model of context-dependent decision-making. **a**, Context-dependent decision-making task. Each trial begins with a brief baseline period (hold). A context cue then briefly appears to indicate either the color or motion context for the current trial (context cue). After a short delay (delay), a sensory stimulus appears that consists of motion and color features (stimulus), and a response can be made at any time. The right motion and red color are associated with the right choice, and the left motion and green color are associated with the left choice. The strength of motion and color stimuli varies from trial to trial as quantified by the motion and color coherence. In the color context, the choice should be made according to the color and ignoring the motion stimulus and vice versa in the motion context. Thus, the same stimulus can map on different responses depending on the context (response; yellow circle). **b**, Architecture of the RNN model. The RNN consists of 50 recurrently connected units, 40 excitatory and 10 inhibitory. The RNN receives six time-varying inputs *u*: two inputs indicating the color and motion context and four inputs representing motion (left and right) and color (red and green) stimuli. We trained the RNN to report its decision by elevating one of two outputs *z*, corresponding to the left versus right choice. **c**, Psychometric functions show that the RNN successfully learns the task; it responds to relevant stimuli and ignores irrelevant stimuli in each context. **d**, RNN connectivity after training appears complex.

We applied our latent circuit inference to RNNs optimized to perform a context-dependent decision-making task, which requires the discrimination of either the color or motion feature of a sensory stimulus depending on the context cue^19^ (Fig. 2a,b and Methods). The RNN successfully learns the task; it makes choices according to the relevant stimulus and ignores the irrelevant stimulus in each context (Fig. 2c). After training, RNN units show heterogeneous mixed selectivity for multiple task variables (Supplementary Fig. 1). The structure in the RNN connectivity responsible for generating the correct behavioral outputs is not immediately obvious (Fig. 2d).

We fitted the latent circuit model to the responses of RNN units and its output behavior during the task. The latent circuit model consisted of eight nodes corresponding to task variables: two context nodes, two sensory color nodes, two sensory motion nodes and two choice nodes. The identity of each node is derived from its input or output, facilitating the interpretation of the latent circuit mechanism. This choice of the latent circuit dimensionality agrees with the dimensionality of RNN responses after training, which is usually close to the total number of inputs and outputs (the first eight principal components accounted for 97.9% of the total variance in RNN responses; Supplementary Fig. 2). The fitted latent circuit model captured an overwhelming amount of variance in the RNN activity (coefficient of determination *r*^2^ = 0.96 on test data) and accurately matched projected RNN trajectories (Supplementary Fig. 3).

**Fig. 3:**
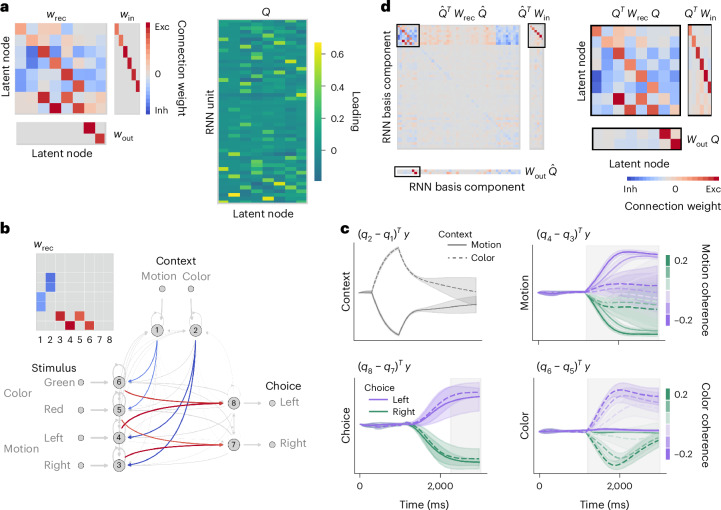
Latent circuit mechanism in the RNN performing a context-dependent decision-making task. **a**, Connectivity matrices of the latent circuit and the embedding matrix *Q* inferred from the responses of the RNN performing the context-dependent decision-making task. **b**, The recurrent connectivity *w*_rec_ in the latent circuit reveals an inhibitory mechanism for context-dependent decision-making. The pattern of excitatory connections from sensory nodes to choice nodes implements two alternative stimulus–response mappings (red arrows in the circuit diagram and red squares in the connectivity matrix). The pattern of inhibitory connections from the context nodes to the sensory nodes implements a suppression mechanism that inhibits the irrelevant stimulus–response mapping in each context (blue arrows in the circuit diagram and blue squares in the connectivity matrix). The schematic of the connectivity matrix (top left) shows only the eight key connections for clarity. The circuit diagram depicts the full latent circuit connectivity in **a**. **c**, Projections of RNN responses onto low-dimensional subspace defined by the columns of embedding *Q*. By construction, the activity along each projection correlates with the activity difference of two nodes in the latent circuit. Projections onto axes corresponding to motion and color nodes reveal suppression of irrelevant stimulus representations. The gray shading indicates the duration of sensory stimulus presentation (motion and color axis) and response period (choice axis). Lines and shaded error bars represent the mean and standard deviation across trials, respectively. **d**, We extend *Q* to an orthonormal basis $\hat{Q}$ for ${{\mathbb{R}}}^{N}$ and transform the RNN connectivity into this basis $\hat{Q}$ (left). The submatrices corresponding to the first *n* = 8 rows and columns (black rectangles, enlarged on the right) closely match the latent circuit connectivity in **a** (correlation coefficient *r* = 0.89).

The inferred recurrent connectivity *w*_rec_ of the latent circuit revealed an interpretable mechanism for context-dependent decision-making (Fig. 3a,b). In the latent circuit, sensory nodes representing stimuli associated with the left choice (left motion and green color) have excitatory connections to the left choice node, and sensory nodes representing stimuli associated with the right choice (right motion and red color) have excitatory connections to the right choice node. This pattern of connections from sensory to choice nodes implements two alternative stimulus–response mappings in the task. Further, the color context node has inhibitory connections to the sensory nodes representing motion, and the motion context node has inhibitory connections to sensory nodes representing color. This pattern of connections from the context nodes to the sensory nodes implements a suppression mechanism that inhibits the irrelevant stimulus–response mapping in each context. Because the irrelevant sensory representation is suppressed, it does not drive the decision output. This suppression mechanism based on inhibition of irrelevant representations is qualitatively similar to mechanisms for context-dependent decision-making hypothesized in previous hand-crafted neural circuit models^8,9^.

**Extended Data Fig. 1:**
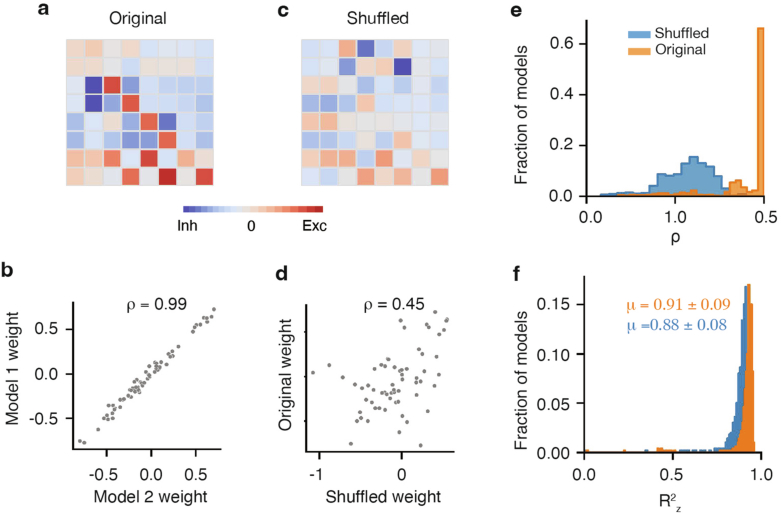
Permutation test for RNNs confirming that the inferred latent connectivity depends on neural responses above the effects of the task. (**a**) Example latent circuit connectivity inferred from original RNN responses. (**b**) Recurrent connectivity weights are highly correlated between two latent circuit models fitted to the same original RNN responses starting from different initialization (Pearson correlation coefficient *ρ* = 0.99). (**c**) Example latent circuit connectivity inferred from shuffled RNN responses. The shuffling procedure randomly permutes neural responses with respect to trial conditions while preserving the input-output relationship on each trial so that the fitted latent circuit models can still perform the task. (**d**) Recurrent connectivity weights are not strongly correlated between the latent circuit model fitted to the shuffled RNN responses (x-axis) and the best model among 500 latent circuit models fitted to the same original RNN responses (y-axis, *ρ* = 0.45). (**e**) Histogram of correlation coefficients between recurrent connectivity weights of the best and all other 499 latent circuit models fitted to the original RNN responses (orange) and the best latent circuit model fitted to the original RNN responses and each of 500 latent circuit models fitted to 500 random permutations of RNN responses (blue). The correlation coefficients are significantly less for the shuffled than original data (one-sided Mann-Whitney U test, *p* < 10^−10^), which indicates that models fitted to shuffled neural responses use more diverse connectivity to perform the task than models fitted to the original data. (**f**) Histogram of task performance metric for latent circuit models in e fitted to original (orange) and shuffled (blue) RNN responses. Latent circuit models fitted to shuffled RNN responses perform the task well and only slightly worse than latent circuit models fitted to the original data (average task performance metric ${r}_{z}^{2}$: original data *μ* = 0.91 ± 0.09, shuffled data *μ* = 0.88 ± 0.08).

We verified that the suppression mechanism revealed in the latent circuit connectivity depended significantly on the RNN responses beyond the constraints imposed by the task alone (Extended Data Fig. 1 and Methods), suggesting that this mechanism reflects the dynamics of the RNN. We therefore proceeded to validate the inferred circuit mechanism directly in the RNN in three ways: in the RNN activity, in the RNN connectivity and by confirming behavioral effects of the RNN perturbations predicted by the latent circuit model.

First, we verified the signatures of the suppression mechanism in the RNN activity. We projected RNN responses onto the columns of *Q*, which represent RNN activity patterns that correlate with the activity of nodes in the latent circuit (Fig. 3c). By projecting RNN responses onto the difference of two columns of *Q* corresponding to the context nodes, we obtain a one-dimensional latent variable correlated with the activity difference of the motion context and color context nodes in the latent circuit. This projection shows RNN trajectories diverging into opposite directions in state space according to context. Next, the choice axis is the difference of two columns of *Q* corresponding to the left and right choice nodes in the latent circuit. Projecting RNN activity onto the choice axis reveals trajectories separating according to choice regardless of context. Further, the motion axis is the difference of columns of *Q* corresponding to the left and right motion nodes, and the color axis is the difference of columns of *Q* corresponding to the red and green color nodes. Projections of RNN activity onto the motion and color axes reveal representations of relevant sensory stimuli, whereas representations of irrelevant stimuli are suppressed. In particular, along the color axis, RNN trajectories separate according to color coherence only on color context trials, whereas on motion context trials, the activity along this axis is suppressed. Similarly, activity along the motion axis is suppressed on color context trials. The suppression of irrelevant sensory representations in RNN activity is consistent with the inhibitory mechanism revealed in the latent circuit connectivity *w*_rec_.

Second, we used the connectivity relationships Eq. (5) to directly validate the latent circuit mechanism in the RNN connectivity. We conjugated the RNN connectivity matrices with the embedding matrix *Q*. The resulting matrices closely match the connectivity in the latent circuit (Fig. 3d; correlation coefficient *r* = 0.89). This agreement confirms that the latent connectivity structure indeed exists in the RNN.

**Fig. 4:**
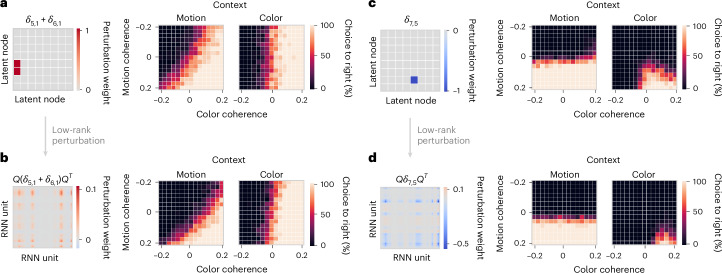
Validating the circuit mechanism via perturbations of RNN connectivity. **a**, Perturbation of the latent circuit connectivity that weakens the inhibitory connection from the motion context node to the sensory nodes representing color (left). This perturbation affects behavior, making the latent circuit sensitive to irrelevant color information, which is visible as a rotation of the decision boundary on motion context trials in the psychometric function (right). **b**, The perturbation in **a** of the latent circuit connectivity maps onto rank-one patterned connectivity perturbation in the RNN (left). This perturbation affects the RNN psychometric function as predicted by the latent circuit model (right). **c**, Perturbation of the latent circuit connectivity that weakens the excitatory connection from the node representing red color to the right choice node (left). The effect of this perturbation on behavior is a decrease in the frequency of right choices on color context trials (right). **d**, Translation of the latent circuit perturbation in **c** onto rank-one perturbation of the RNN connectivity (left) confirms the predicted behavioral effect in the RNN (right).

Finally, to ultimately validate that this latent connectivity structure supports the behavioral task performance, we tested whether patterned perturbations of the RNN connectivity (Eq. (6)) produced the same behavioral effects as predicted by the latent circuit model. We consider two perturbations designed to test the inhibitory mechanism. The first perturbation ‘turns off’ the context mechanism by weakening the inhibitory connections from a context node to sensory nodes representing irrelevant stimuli in that context (Fig. 4a). In the RNN, this perturbation maps onto a rank-one change in the recurrent connectivity (Fig. 4b). The latent circuit mechanism predicts that weakening the inhibitory connections from the motion context node to sensory nodes representing color would make the circuit sensitive to the irrelevant color information on motion context trials. Indeed, weakening these connections in the latent circuit produced the predicted behavioral effect in the psychometric function, visible as a rotation of the decision boundary on motion context trials (Fig. 4a). Perturbations of the RNN connectivity along the corresponding pattern produced similar behavioral effects (Fig. 4b), confirming that this connectivity pattern implements suppression of irrelevant sensory representations in the RNN.

The second perturbation ‘turns off’ one of the stimulus–response mappings by weakening the excitatory connection from a sensory node to a choice node. The latent circuit mechanism predicts that weakening the excitatory connection from the red color node to the right choice node (Fig. 4c) would impair the network’s ability to make right choices on the color context trials. Weakening this connection in the latent circuit indeed decreased the frequency of right choices on color context trials (Fig. 4c). This perturbation maps onto a rank-one connectivity perturbation in the RNN, which produced similar behavioral effects (Fig. 4d). This result confirms both the behavioral relevance of the latent sensory representation and the excitatory mechanism by which it drives choices in the RNN.

Together, these results confirm that the RNN uses the suppression mechanism in which context representations inhibit irrelevant sensory representations. This mechanism is reflected in the low-dimensional dynamics revealed by projecting RNN activity onto axes defined by the latent circuit embedding *Q*. We identified this mechanism as a latent low-dimensional structure in the RNN connectivity and ultimately validated it by confirming behavioral effects of the RNN connectivity perturbations.

### Space of latent circuit mechanisms

We next asked whether RNNs trained on the same task arrive at different circuit solutions for context-dependent decision-making. The latent circuit inference enables us to determine whether different RNNs use the same circuit mechanism. Although two RNNs trained on the same task may have distinct high-dimensional connectivity, the latent circuit inference can reveal whether these RNNs use similar low-dimensional connectivity structure to generate task-relevant dynamics. Therefore, we assessed the similarity of task solutions in RNNs by comparing their low-dimensional latent connectivity.

To explore the space of circuit mechanisms, we trained an ensemble of 200 RNNs with randomly initialized connectivity to the same level of task performance (*r*^2^ = 0.93 ± 0.01, coefficient of determination for the RNN and target outputs, mean ± s.d. across networks; Supplementary Fig. 4). For each of these RNNs, we fitted an ensemble of 100 latent circuit models starting with random initializations of the parameters for latent connectivity and embedding *Q* and selected 10 latent circuits with the highest fit quality on the test data, which formed the set of converged solutions (Methods). All converged latent circuits provided accurate fits of the RNN responses (Supplementary Fig. 4).

**Fig. 5:**
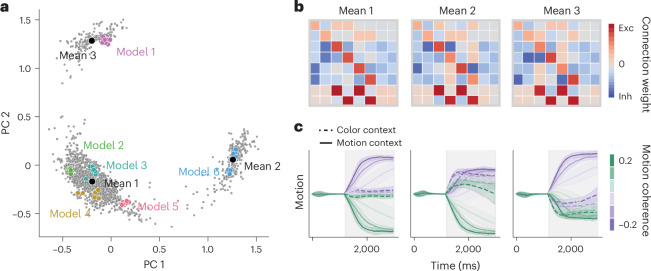
Space of latent circuit mechanisms for context-dependent decision-making in RNNs. **a**, Projection onto the first two principal components (PC) of the set of recurrent connectivity matrices in latent circuits obtained from 200 RNNs trained to perform the context-dependent decision-making task. For each of these RNNs, we fitted an ensemble of 100 latent circuits with random initializations and selected 10 latent circuits with the best fit quality on test data to form the set of converged solutions (gray dots). Latent circuits fitted to responses of a single RNN fall within close proximity of each other (colored dots). The projection reveals three major solution clusters, which we partitioned by fitting data with a Gaussian mixture model with three components. The majority of circuits fell within a single cluster, whereas relatively fewer circuits fell within one of two other clusters. **b**, Recurrent connectivity matrices corresponding to the means of three clusters in **a**. **c**, Dynamics of the activity difference between two nodes representing the motion stimulus in latent circuits with the mean connectivity matrices in **b**. These dynamics reveal asymmetric representations of stimuli in two of the clusters (center and right). Lines and shaded error bars represent the mean and standard deviation across trials, respectively. Source data

To visualize the space of latent circuit solutions, we applied a principal component analysis to the flattened connectivity matrices *w*_rec_ and projected the data onto the first two principal components, which accounted for 42% of total variance (Fig. 5a). The converged latent circuits fitted to the responses of a single RNN fell within a close proximity of each other (Fig. 5a), and their connectivity was highly correlated (*r* = 0.98 ± 0.02, mean correlation coefficient between connectivity weights of the best and other converged circuits, mean ± s.d. across RNNs; Methods), which indicates the uniqueness of the latent circuit mechanism in each particular network. The variability of task solutions was much greater across RNNs; the variance of latent connectivity across all RNNs was about four times the average variance in latent connectivity across multiple fits of a single RNN (variance ratio of 4.3).

**Extended Data Fig. 2:**
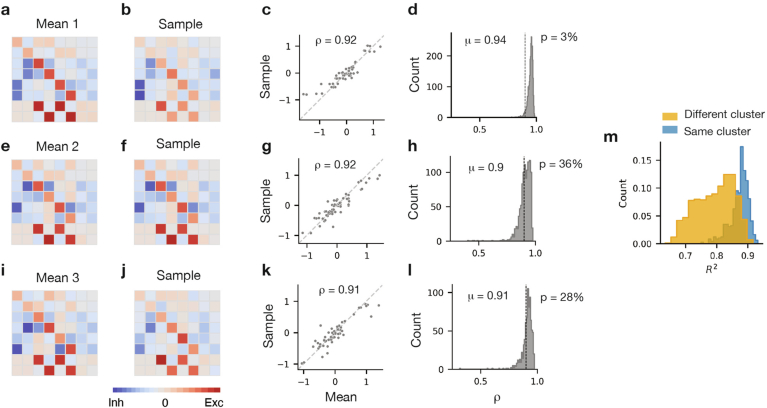
Similarity of task solutions within clusters. For the ensemble of RNNs (Fig. 5 in the main text), we quantified the similarity of task solutions within each cluster by computing correlation coefficients between the latent connectivity of all RNNs within a cluster and the mean connectivity of that cluster. (**a**) Mean latent connectivity for cluster 1. (**b**) Latent connectivity of a sample model from cluster 1. (**c**) Scatter plot of weights in the mean connectivity shown in a (x-axis) versus sample connectivity shown in b (y-axis). The correlation coefficient is *ρ* = 0.92. (**d**) Distribution of correlation coefficients between the mean connectivity of cluster 1 and all latent models in cluster 1. High average correlation coefficient *μ* = 0.94 indicates that task solutions within the cluster are similar. Only *p* = 3% of RNNs in cluster 1 have correlation coefficient with the cluster mean less than 0.9. (**e**)-(**h**) Same as a-d for cluster 2. (**i**)-(**l**) Same as a-d for cluster 3. (**m**) Different inferred task solutions across clusters result from differences in neural responses across RNNs. We randomly selected 1,500 latent circuit models from the ensemble and fitted each circuit to responses of a randomly selected target RNN. During fitting, we only optimized the embedding matrix *Q* while holding the latent circuit connectivity fixed. Fits were significantly worse when the latent circuit and the target RNN were sampled from different clusters (orange) than the same cluster (blue, one-sided Mann-Whitney U test: *U* = 52, 593, *p* < 10^−10^).

The latent circuit solutions from all RNNs formed three major clusters. RNNs in all clusters had similar task performance (Supplementary Fig. 5). The correlation coefficients between the latent connectivity of all RNNs within a cluster and the mean connectivity of that cluster was high (mean correlation coefficients: *r* = 0.94 cluster 1, *r* = 0.9 cluster 2 and *r* = 0.91 cluster 3; Extended Data Fig. 2), which indicates that the RNNs within each cluster had a similar circuit mechanism. To test whether circuit mechanisms varied between RNNs from different clusters, we sampled latent circuits randomly from the ensemble and fitted these circuits to responses of randomly sampled RNNs, optimizing only the embedding matrix *Q* while holding the latent circuit connectivity fixed. These latent models produced a significantly worse fit when the latent circuit and the target RNN were sampled from different clusters than from the same cluster (Extended Data Fig. 2m; one-sided Mann–Whitney *U*-test, *U* = 52, 593, *P* < 10^−10^), confirming differences in circuit mechanisms across clusters. This result further reinforces that task alone does not uniquely determine the circuit mechanism, and not any connectivity sufficient to perform the task can account for a specific set of neural responses in an RNN.

**Extended Data Fig. 3:**
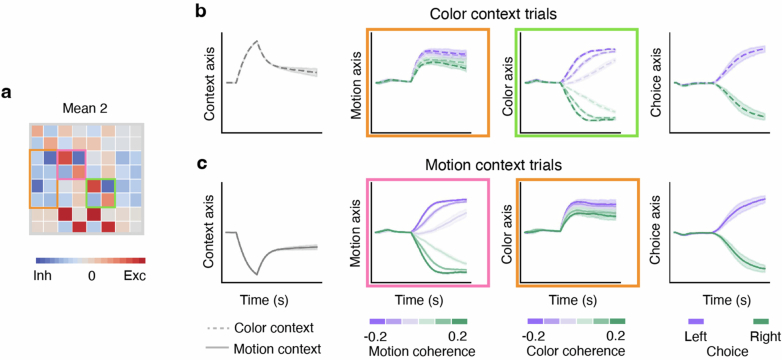
Asymmetric inhibitory mechanism for context-dependent decision-making. In the ensemble of 200 RNNs trained on the context-dependent decision-making task, latent circuit solutions form three major clusters (Fig. 5 in the main text). In clusters 2 and 3, the latent circuit connectivity and dynamics show asymmetry with a bias towards the right or left stimulus representation. Yet, these circuits operate via the same inhibitory mechanism based on suppression of irrelevant stimuli. (**a**) Mean recurrent connectivity matrix for RNNs in cluster 2. Context nodes have stronger inhibitory connections onto sensory nodes that provide evidence for the right choice than onto sensory nodes that provide evidence for the left choice (orange rectangle). This bias is counterbalanced by asymmetry in the recurrent connections between sensory nodes. The motion-right node has stronger self-excitation than the motion-left node, and the motion-right node stronger inhibits the motion-left node than vice versa (pink square). The recurrent connections between the color-right and color-left nodes show the same asymmetry (green square). Thus, the recurrent interactions between sensory nodes are biased in favor of the right evidence to counterbalance the bias in inhibitory connections from contextual nodes which favor the left evidence. (**b**) RNN responses projected onto subspace defined by columns of the embedding matrix *Q* of the latent circuit model, averaged over all RNNs in cluster 2. The responses are shown on color context trials, when the motion stimulus is irrelevant. The projections show that the irrelevant motion responses are not suppressed but shifted toward the left-motion representation (orange box), due to stronger inhibition of the motion-right node by the color context node. At the same time, the relevant color responses are shifted toward the color-right representation (green box), due to biased recurrent connectivity between sensory color nodes. Thus, irrelevant motion responses provide a bias in favor of the left choice, but this bias is compensated by the bias in relevant color responses favoring the right choice. As a result, the RNN performance is unbiased (Supplementary Fig. 5). On trials when both color and motion evidence points to the left choice, the irrelevant motion responses reinforce the relevant color responses. Lines and shaded error bars represent the mean and standard deviation across trials, respectively. (**c**) Same as b but for motion context trials. Irrelevant color responses provide a bias in favor of the left choice (orange box), but this bias is compensated by the bias in relevant motion responses favoring the right choice (pink box). RNNs in cluster 3 operate via a similar asymmetric inhibitory mechanism with the flipped biases for the left and right stimulus representations. Lines and error bars represent the mean and one standard deviation computed across trials.

To understand how the circuit mechanisms vary across clusters, we examined the mean latent connectivity matrix of each cluster. The mean connectivity matrices revealed circuits that all showed signatures of the suppression mechanism, with context nodes inhibiting irrelevant sensory nodes (Fig. 5b). In the main cluster, the circuits were balanced and symmetric, with approximately equal strength of excitation or inhibition between nodes representing different contexts and stimulus–response mappings. This balance was reflected in the dynamics and representations of stimuli (Fig. 5c). In the two other clusters, the circuits showed asymmetry in connectivity, with stronger inhibition from context to some sensory nodes counterbalanced by stronger self-excitation for these sensory nodes. These asymmetries were consistently reflected in dynamics and the representations of stimuli, which showed a bias toward the left or right stimulus representations depending on the cluster. Although the circuit solutions in two of the clusters exploit asymmetries in the representations of sensory evidence, they still operate by an inhibitory mechanism in which irrelevant responses are suppressed (Extended Data Fig. 3).

**Extended Data Fig. 4:**
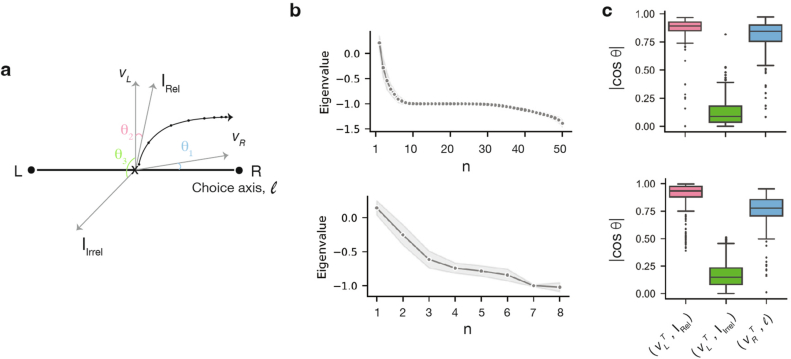
Dynamical selection vector mechanism in RNNs and latent circuits that implement the inhibitory suppression mechanism. (**a**) Schematic of the dynamical selection vector mechanism, which describes context-dependent decision-making in terms of linearized RNN dynamics in each context^19^. In this mechanism, choice is integrated along a line attractor in each context (*l*, choice axis). RNN dynamics linearized at a point *x* along this axis are controlled by the left and right eigenvectors for the zero eigenvalue. Only inputs aligned with the left eigenvector (*v*_*L*_, selection vector) drive activity along the right eigenvector (*v*_*R*_), which has a non-zero projection onto the choice axis *l*. Inputs not aligned with the selection vector are not integrated along the choice axis. Context-dependent selection arises because in each context, the relevant sensory input (*I*_Rel_) aligns with the selection vector and the irrelevant sensory input (*I*_Irrel_) does not. In each context, the selection mechanism is characterized by three angles determining the alignment between the choice axis and the right eigenvector (*θ*_1_), the selection vector and the relevant input (*θ*_2_), and the selection vector and the irrelevant input (*θ*_3_). Schematic shows this arrangement in one context. (**b**) We analyzed the dynamical mechanism in an ensemble of 200 RNNs trained on a context-dependent decision-making task (upper panel) and latent circuits inferred from their responses (lower panel, the best-fitting circuit for each RNN). These RNNs and latent circuits use the inhibitory suppression mechanism to perform the task (Fig. 5 in the main text). To find the choice axis, we fit a linear regression to neural responses. The regression predicted the neural response at the last time point of each trial as a linear combination of the context, motion coherence, color coherence and choice variables for that trial. The choice axis is a vector comprising regression coefficients for choice from all neurons. In each context, we then find the point *x* corresponding to the linear regression prediction with the choice, motion, and color variables set to zero, and context variable set to either 1 or − 1. We then compute eigenvalues and their corresponding left and right eigenvectors for the recurrent dynamics linearized at *x*. In all models, the top eigenvalue was close to zero and the real parts of the remaining eigenvalues were negative, consistent with an approximate line attractor. Dots are the mean and shading is standard deviation across models and two contexts. (**c**) We take *v*_*L*_ and *v*_*R*_ to be the left and right eigenvectors for the top eigenvalue. We quantify the arrangement of these eigenvectors relative to the relevant and irrelevant inputs and choice axis by computing the absolute value of the cosine of the angles *θ*_1_, *θ*_2_, *θ*_3_ between these vectors in all 200 RNNs (upper panel) and their latent circuits (lower panel). Consistent with the selection-vector mechanism, *v*_*R*_ has non-zero overlap with the choice axis (blue), and the relevant input has higher overlap with the selection vector *v*_*L*_ (pink) than the irrelevant input (green) both in RNNs and latent circuits. The center line of the box-and-whisker plot marks the median, the box extends from the 25th (*Q*_1_) to the 75th (*Q*_3_) percentiles, and the whiskers extend to 1.5 times the interquartile range (*Q*_3_ − *Q*_1_). Dots indicate data points that lie outside this range.

**Extended Data Fig. 5:**
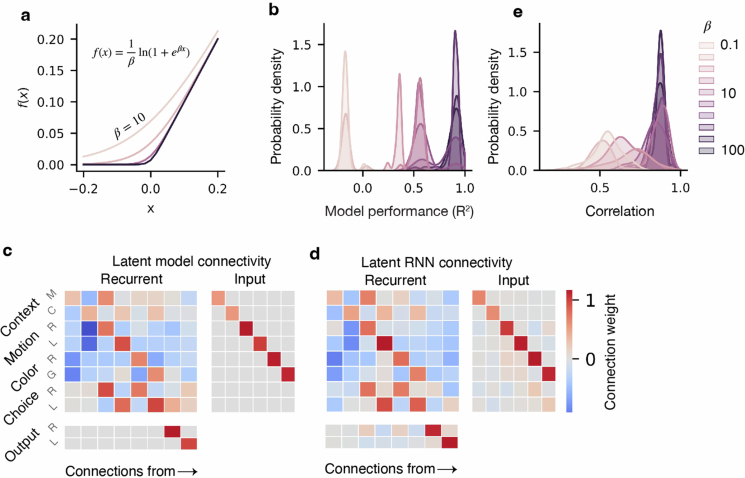
Latent circuit models for RNNs with Softplus non-linearity. (**a**) The shape of the Softplus activation function is controlled by a parameter *β*. As *β* increases, the activation function converges to a rectified-linear (ReLU) non-linearity. (**b**) We trained RNNs with the Softplus activation function of units to perform the context-dependent decision-making task, using different values of the parameter *β* across RNNs (25 RNNs for each value of *β*). Only models with sufficiently large *β* (*β* > 10) learn to perform the task successfully. (**c**) Example connectivity of a latent circuit model with ReLU non-linearity fitted to responses of an RNN with Softplus non-linearity for *β* = 10. The latent connectivity reveals a similar inhibitory mechanism as found in RNNs with the ReLU activation function. (**d**) Conjugating the RNN connectivity matrix with the embedding matrix *Q* of the latent circuit model reveals a low-dimensional structure *Q*^*T*^*W*_rec_*Q* that closely matches the latent circuit connectivity *w*_rec_ in c (Pearson correlation coefficient *ρ* = 0.88). Thus, the latent circuit model identifies the inhibitory mechanism in the RNN connectivity, despite the RNN and latent circuit model have different non-linearity. (**e**) Distribution of correlation coefficients *ρ* between the latent circuit connectivity *w*_rec_ and the low-dimensional connectivity structure *Q*^*T*^*W*_rec_*Q* in RNN, for all RNNs with different *β* in b. The correlation is strong for all RNNs that successfully perform the task (for *β* > 10).

**Extended Data Fig. 6:**
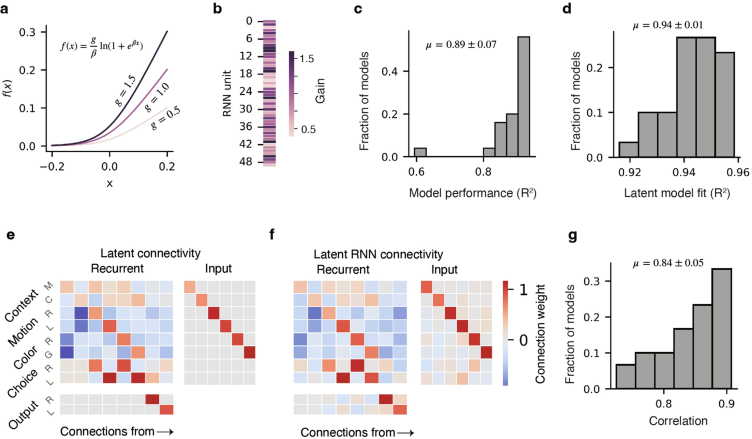
Latent circuit models for RNNs with Softplus non-linearity and heterogeneous gains across units. (**a**) The shape of the Softplus activation function for *β* = 10 and different values of a multiplicative gain parameter *g*. (**b**) The gain parameter was randomly selected for each RNN unit from the interval [0.5, 1.5]. Example vector of gain parameters for an RNN model is shown. (**c**) We trained 25 RNNs with the Softplus activation function (*β* = 10) and heterogeneous gains across units to perform the context-dependent decision-making task. Most RNNs achieved high performance level on the task. (**d**) The latent circuit models with ReLU non-linearity provided a good fit to responses of these RNNs. Distribution of the fit quality metric *R*^2^ across RNNs is shown. (**e**) Example connectivity of a latent circuit model with ReLU non-linearity fitted to responses of an RNN with Softplus non-linearity and heterogeneous gains. The latent connectivity reveals a similar inhibitory mechanism as found in RNNs with the ReLU activation function. (**f**) Conjugating the RNN connectivity matrix with the embedding matrix *Q* of the latent circuit model reveals a low-dimensional structure *Q*^*T*^*W*_rec_*Q* that closely matches the latent circuit connectivity *w*_rec_ in e (Pearson correlation coefficient *ρ* = 0.88). Thus, the latent circuit model identifies the inhibitory mechanism in the RNN connectivity, despite the architecture mismatch between the RNN and latent circuit model. (**g**) Distribution of correlation coefficients *ρ* between the latent circuit connectivity *w*_rec_ and the low-dimensional connectivity structure *Q*^*T*^*W*_rec_*Q* in RNN, for all RNNs in c. Strong correlations indicate that the latent circuit connectivity faithfully represents the connectivity in RNNs despite their architecture mismatch.

The inhibitory suppression mechanism that we consistently found across RNNs may seem distinct from a dynamical selection vector mechanism previously identified in RNNs trained on a similar task, which apparently does not require suppression of irrelevant sensory responses^19^. We analyzed dynamics in our RNNs and found the same selection vector mechanism (Extended Data Fig. 4), indicating that the dynamical selection vector mechanism is a local linear description of the inhibitory circuit mechanism. Furthermore, we found the same inhibitory suppression mechanism in RNNs trained without constraining their inputs to be orthogonal (Supplementary Fig. 6) and in RNNs with different biologically plausible nonlinearities (Extended Data Figs. 5 and 6). Thus, the space of latent circuit solutions found by all our RNN models of context-dependent decision-making can be characterized by a common suppression mechanism.

### Representations of irrelevant stimuli in the PFC

Our finding that RNNs use the inhibitory mechanism for context-dependent decision-making appears in conflict with previous work, which suggested that in both RNNs and the PFC, irrelevant sensory responses are not significantly suppressed^19,21,51^. This conclusion was derived using dimensionality reduction methods that fit neural responses with regression models^19,21,41–43^ to find low-dimensional projections that best correlate with task variables (Fig. 1b). In these projections, task variables do not interact but are demixed in orthogonal dimensions. By contrast, representations of task variables in the latent circuit model interact via recurrent connectivity to implement the computations necessary to solve the task. We therefore sought to determine whether the latent circuit and regression models identify different task representations in the same PFC responses.

**Extended Data Fig. 7:**
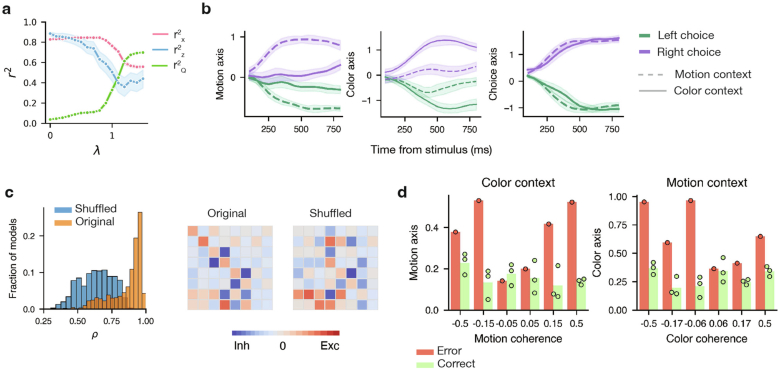
Latent circuit analysis of PFC recordings from monkey A. (**a**) When fitting the latent circuit model to PFC data, the hyperparameter *λ* controls the balance between variance explained by the task-relevant subspace ${r}_{Q}^{2}$, the fits between projected PFC responses and the latent circuit trajectories in this subspace ${r}_{x}^{2}$, and the performance of the latent circuit on the task ${r}_{z}^{2}$. The terms ${r}_{Q}^{2}$ and ${r}_{x}^{2}$ both decrease with *λ*, whereas the term ${r}_{Q}^{2}$ increases. We choose *λ* = 0.5, since at this value, ${r}_{Q}^{2}$ and ${r}_{x}^{2}$ are simultaneously maximized under the constraint that the latent circuit model still has high task performance (${r}_{z}^{2} \sim 80 \%$). We obtained similar results for *λ* = 0.4 and *λ* = 0.6. Points represent mean across 200 fits. Shading represents a 95% confidence interval estimated via a bootstrap with 1,000 resamples. (**b**) Projection of PFC responses onto motion, color and choice axes obtained from the latent circuit model. Trials are sorted by the monkey’s choice and context. These projections confirm that the motion and color axes in the latent circuit model do not represent choice on all trials and that the observed suppression of irrelevant representations (Fig. 6a in the main text) does not arise from sorting choice activity by irrelevant stimulus coherence. Lines and shaded error bars represent the mean and standard deviation across trials, respectively. (**c**) Permutation test confirming that the inferred latent connectivity depends on PFC responses above the effects of the task. Example latent circuit connectivity inferred from the original PFC responses (center) and from shuffled PFC responses permuted with respect to trial conditions while preserving the input-output relationship on each trial so that the latent model can perform the task (right). Histogram of correlation coefficients *ρ* between recurrent connectivity weights of the best and all other 999 latent circuit models fitted to the original PFC responses (orange) and the best latent circuit model fitted to the original PFC responses and each of 1,000 latent circuit models fitted to 1,000 random permutations of PFC responses (blue). The correlation coefficients are significantly less for the shuffled than original data (one-sided Mann-Whitney U test, *p* < 10^−10^), which indicates that PFC responses significantly constrain the inferred latent connectivity above the effect of the task. (**d**) Representation of irrelevant stimuli on error trials. The absolute value of the projection onto motion axis (left) of PFC responses on incongruent color-context trials sorted by motion coherence (x-axis) and whether the animal makes a correct choice (green) or error (red). The irrelevant motion representation is significantly less suppressed (has higher amplitude) on error than correct trials (combined condition one-sided Mann-Whitney test, *p* = 0.0064, *U* = 0.31, *n* = 24). This effect is largest when the irrelevant motion evidence is strongest. We see the same effects in the absolute value of the projection onto the color axis (right) of PFC responses on incongruent motion-context trials sorted by color coherence (x-axis) and whether the animal makes a correct choice (green) or error (red). The irrelevant color representation is significantly less suppressed on error than correct trials (combined condition one-sided Mann-Whitney test, *p* = 0.0002, *U* = 0.35, *n* = 24). These effects were not due to a non-specific increase in activity on error trials, as the representations of relevant stimuli were significantly smaller on error than correct trials (one-sided condition-combined Mann-Whitney test: color axis in color context *p* = 0.0024, *U* = 0.35, *n* = 12, motion axis in motion context *p* = 0.045, *U* = 0.29, *n* = 12). Dots represent individual trial conditions, bars represent their mean.

**Extended Data Fig. 8:**
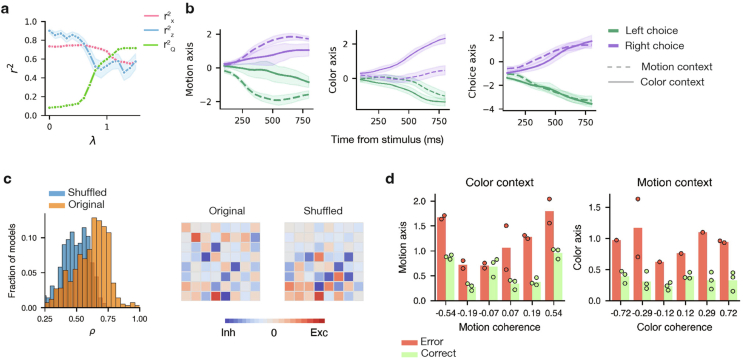
Latent circuit analysis of PFC recordings from monkey F. (**a**) When fitting the latent circuit model to PFC data, the hyperparameter *λ* controls the balance between variance explained by the task-relevant subspace ${r}_{Q}^{2}$, the fits between projected PFC responses and the latent circuit trajectories in this subspace ${r}_{x}^{2}$, and the performance of the latent circuit on the task ${r}_{z}^{2}$. The terms ${r}_{Q}^{2}$ and ${r}_{x}^{2}$ both decrease with *λ*, whereas the term ${r}_{Q}^{2}$ increases. We choose *λ* = 0.5, since at this value, ${r}_{Q}^{2}$ and ${r}_{x}^{2}$ are simultaneously maximized under the constraint that the latent circuit model still has high task performance (${r}_{z}^{2} \sim 82 \%$). We obtained similar results for *λ* = 0.4 and *λ* = 0.6. Points represent mean across 200 fits. Shading represents a 95% confidence interval estimated via a bootstrap with 1,000 resamples. (**b**) Projection of PFC responses onto motion, color and choice axes obtained from the latent circuit model. Trials are sorted by the monkey’s choice and context. These projections confirm that the motion and color axes in the latent circuit model do not represent choice on all trials and that the observed suppression of irrelevant representations (Extended Data Fig. 9a) does not arise from sorting choice activity by irrelevant stimulus coherence. Lines and shaded error bars represent the mean and standard deviation across trials, respectively. (**c**) Permutation test confirming that the inferred latent connectivity depends on PFC responses above the effects of the task. Example latent circuit connectivity inferred from the original PFC responses (center) and from shuffled PFC responses permuted with respect to trial conditions while preserving the input-output relationship on each trial so that the latent model can perform the task (right). Histogram of correlation coefficients *ρ* between recurrent connectivity weights of the best and all other 999 latent circuit models fitted to the original PFC responses (orange) and the best latent circuit model fitted to the original PFC responses and each of 1,000 latent circuit models fitted to 1,000 random permutations of PFC responses (blue). The correlation coefficients are significantly less for the shuffled than original data (one-sided Mann-Whitney U test, *p* < 10^−10^), which indicates that PFC responses significantly constrain the inferred latent connectivity above the effect of the task. (**d**) Representation of irrelevant stimuli on error trials. The absolute value of the projection onto motion axis (left) of PFC responses on incongruent color-context trials sorted by motion coherence (x-axis) and whether the animal makes a correct choice (green) or error (red). The irrelevant motion representation is significantly less suppressed (has higher amplitude) on error than correct trials (combined condition one-sided Mann-Whitney test, *p* = 0.0002, *U* = 0.33, *n* = 30). This effect is largest when the irrelevant motion evidence is strongest. We see the same effects in the absolute value of the projection onto the color axis (right) of PFC responses on incongruent motion-context trials sorted by color coherence (x-axis) and whether the animal makes a correct choice (green) or error (red). The irrelevant color representation is significantly less suppressed on error than correct trials (combined condition Mann-Whitney test, *p* = 0.0002, *U* = 0.375, *n* = 26). These effects were not due to a non-specific increase in activity on error trials, as the representations of relevant stimuli were significantly smaller on error than correct trials (one-sided condition-combined Mann-Whitney test: color axis in color context *p* = 0.0002, *U* = 0.36, *n* = 24, motion axis in motion context *p* = 0.0002, *U* = 0.38, *n* = 20). Dots represent individual trial conditions, bars represent their mean.

We fitted the latent circuit model to the same dataset of PFC recordings during context-dependent decision-making as in the previous studies^19,21^. The dataset consists of several hundred PFC neurons (*n* = 727 and 574 for monkeys A and F, respectively) recorded from two rhesus monkeys performing a context-dependent decision-making task^19^ (Fig. 2a). We fitted latent circuit models to smoothed condition-averaged PFC responses during a 750-ms window starting 100 ms after the stimulus onset^19^ (Methods). The latent circuit model provided good fits of PFC responses projected onto the low-dimensional subspace spanned by the columns of the inferred embedding matrix *Q* for both monkeys (*r*^2^ = 0.88 and 0.76 on test data for monkeys A and F, respectively; Extended Data Figs. 7a and 8a). The task subspace *Q* explained a smaller fraction of total variance in the PFC responses (11.0% and 7.0% for monkeys A and F, respectively) than in RNNs (Supplementary Fig. 2), which is comparable to previous reports of task-relevant variance in the PFC^42^ and reflects the high dimensionality of PFC activity^19^.

**Fig. 6:**
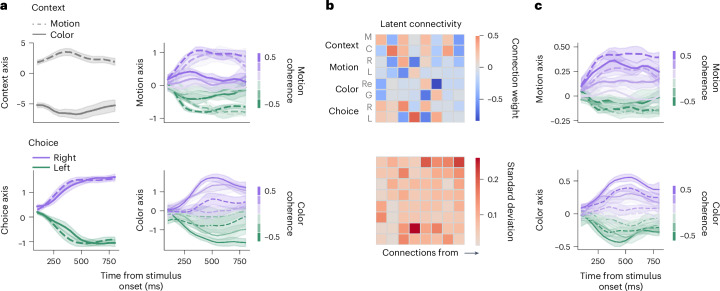
Representations of irrelevant stimuli in the PFC during context-dependent decision-making. **a**, Projection of PFC responses from monkey A onto task subspace defined by the columns of the embedding matrix *Q* in the latent circuit model fitted to the PFC data. The four latent circuit axes correspond to context, choice, motion and color representations, as in Fig. 3c. Projections onto motion and color axes reveal that representations of stimuli in the PFC are suppressed when they are irrelevant. Lines and shaded error bars represent the mean and standard deviation across trials, respectively. **b**, Latent circuit connectivity inferred from PFC responses (top) shows an inhibitory mechanism similar to that observed in RNNs (Fig. 5b). The checkerboard pattern of connections from sensory nodes to choice nodes implements the alternative stimulus–response mappings. The pattern of inhibitory connections from context nodes to sensory nodes implements a suppression mechanism, which inhibits the irrelevant stimulus–response mapping in each context. The standard deviation of connectivity weights across the top ten latent circuit fits to the same PFC data quantifies the estimation uncertainty for each connection (bottom); M, motion; C, color; R, right; L, left; Re, red; G, green. **c**, The latent circuit model in which the latent recurrent connectivity matrix is constrained to be 0 (*w*_rec_ ≡ 0) identifies a different task subspace in which projections of PFC responses onto motion and color axes show little suppression of motion and color representations on trials when they are irrelevant, reproducing results from previous studies^19,21^. Lines and shaded error bars represent the mean and standard deviation across trials, respectively. Source data

**Extended Data Fig. 9:**
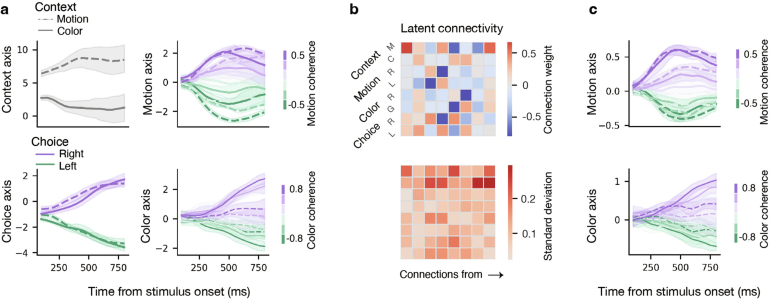
Representations of irrelevant stimuli in PFC recordings from monkey F. (**a**) Projection of PFC responses from monkey F onto task-subspace defined by the columns of the embedding matrix *Q* in the latent circuit model fitted to the PFC data. The four latent circuit axes correspond to context, choice, motion and color representations as in Fig. 6a in the main text. Projections onto motion and color axes reveal that representations of stimuli in PFC are suppressed when they are irrelevant. Less suppression along the motion axis on color context trials (in comparison to Monkey A and the color axis for Monkey F) is consistent with behavior: psychometric functions show Monkey F choices are sensitive to irrelevant motion information^19^. Lines and shaded error bars represent the mean and standard deviation across trials, respectively. (**b**) Latent circuit connectivity inferred from PFC responses (upper panel) shows an inhibitory mechanism similar to that observed in monkey A (cf. Fig. 6b in the main text). The checkerboard pattern of connections from sensory to choice nodes implements the alternative stimulus-response mappings. The pattern of inhibitory connections from the context to sensory nodes implements a suppression mechanism which inhibits the irrelevant stimulus-response mapping in each context. Standard deviation of connectivity weights across top 10 latent circuit fits to the same PFC data quantifies the estimation uncertainty for each connection (lower panel). (**c**) The latent circuit model in which the latent recurrent connectivity matrix is constrained to be zero (*w*_rec_ ≡ 0) identifies a different task-subspace, in which projections of PFC responses onto motion axes show less suppression of motion representations on trials when they are irrelevant, reproducing results from previous studies^19^. Projections onto the color axis show suppressed color representations on motion context trials, consistent with results from targeted dimensionality reduction applied to the same data^19^, and may reflect a lack of recorded color-sensitive neurons which are unmodulated by context. Lines and shaded error bars represent the mean and standard deviation across trials, respectively. Source data

**Extended Data Fig. 10:**
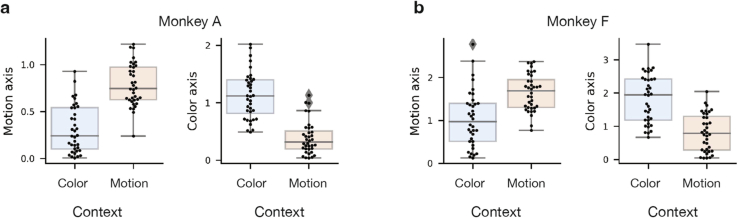
Statistically significant suppression of irrelevant stimulus representations in PFC. (**a**) Suppression of irrelevant stimulus representations in monkey A. Distribution across trials of the absolute value of the projection of the PFC activity at the last time bin of stimulus presentation (*t* = 800 ms in Fig. 6 in the main text) onto motion (left) and color axes (right) of the latent circuit model, separately for color (blue) and motion context trials (red). The distributions include all correct trials for all combinations of motion and color coherence. The amplitude of stimulus representation along these axes is significantly smaller when stimulus is irrelevant than when it is relevant (one-sided Mann-Whitney U test, motion axis *p* = 1.4 ⋅ 10^−9^, color axis *p* = 1.0 ⋅ 10^−10^, *n* = 36 trial conditions per context). The center line of the box-and-whisker plot marks the median, the box extends from the 25th (*Q*_1_) to the 75th (*Q*_3_) percentiles, and the whiskers extend to 1.5 times the interquartile range (*Q*_3_ − *Q*_1_). Diamonds indicate points which lie outside this range. (**b**) Same as a for monkey F (motion axis *p* = 1.5 ⋅ 10^−5^, color axis *p* = 1.6 ⋅ 10^−7^, *n* = 36 trial conditions per context).

Similar to the RNNs, projecting PFC responses onto the axes identified by the latent circuit model revealed significant suppression of stimulus representations when they were irrelevant (Fig. 6a and Extended Data Figs. 9a and 10; one-sided Mann–Whitney *U*-test; monkey A: motion *P* = 1.4 × 10^−9^ and *U* = 120, color *P* = 10^−10^ and *U* = 83; monkey F: motion *P* = 1.5 × 10^−5^ and *U* = 277, color *P* = 1.6 × 10^−7^ and *U* = 194; *n* = 36). This suppression was not due to correlation of activity along the motion and color axes with choice (Extended Data Figs. 7b and 8b). Consistent with the suppression seen in projected PFC activity, the inferred latent circuit connectivity showed inhibitory connections from context nodes to sensory nodes representing irrelevant stimuli in each context (Fig. 6b and Extended Data Fig. 9b). We confirmed that PFC responses significantly constrained the inferred latent connectivity above the effect of the task (Extended Data Figs. 7c and 8c and Methods), indicating that the suppression mechanism reflects dynamics in the PFC data. The fact that the latent circuit model correctly performs the task and accurately fits projected PFC responses indicates that the suppression seen in the PFC data is sufficient to produce the context-dependent decision-making behavior.

The latent circuit model identified a subspace in PFC activity in which representations of irrelevant stimuli were suppressed, whereas regression methods uncovered subspaces in the same PFC data in which stimuli were nearly equally represented across contexts^19,21^. To confirm that this difference results from recurrent interactions among task variables in the latent circuit model, we fitted PFC data with a modified latent circuit model in which the latent recurrent connectivity was constrained to be 0. In this model, like in regression models, the latent variables do not causally influence each other. Indeed, this model found low-dimensional subspaces in which irrelevant sensory representations were less suppressed in both monkeys (Fig. 6c and Extended Data Fig. 9c), consistent with previous studies^19,21^. These subspaces explained a smaller amount of total variance in PFC responses (0.6% and 1.1% for monkeys A and F, respectively) than the latent circuit model. Thus, it is possible to find both types of representations in the PFC, and the question arises about which of these possible representations are causally linked to behavior.

Although we cannot directly assess the behavioral relevance of different representations in the PFC via perturbations, we can test the inhibitory suppression mechanism using neural activity on error trials. Specifically, the suppression mechanism predicts that the representations of irrelevant stimuli (along the motion and color axes identified by the latent circuit model) should be less suppressed on error than correct trials. We tested this prediction on incongruent trials on which the relevant and irrelevant stimuli point to opposite choices (Methods) because the errors on incongruent trials are more likely to result from a failure of the contextual mechanism than other factors (for example, attention lapse). The prediction was clearly borne out by the data; in both contexts for both monkeys, the irrelevant representations were significantly less suppressed on error than correct trials (Extended Data Figs. 7d and 8d; combined condition Mann–Whitney *U*-test; monkey A: *P* = 0.0086, *U* = 0.313 and *n* = 24 on color context trials and *P* = 0.0002, *U* = 0.354 and *n* = 24 on motion context trials; monkey F: *P* = 0.0002, *U* = 0.333 and *n* = 26 on color context trials and *P* < 10^−10^, *U* = 0.375 and *n* = 30 on motion context trials), suggesting that the representations identified by the latent circuit model in PFC activity are related to the behavioral task execution.

### Behavioral relevance of low-dimensional representations

**Fig. 7:**
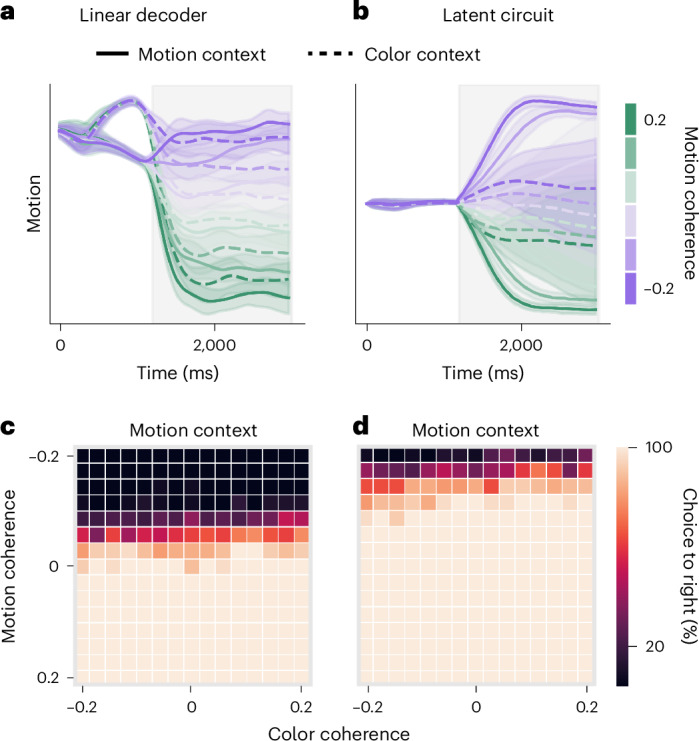
Representations of irrelevant stimuli in the RNN do not drive behavior. **a**, Projection of RNN responses onto the decoder axis reveals an equal representation of motion coherence on both color context and motion context trials. Lines and shaded error bars represent the mean and standard deviation across trials, respectively. **b**, Projection of the same RNN responses onto the motion axis from the latent circuit model reveals a representation of motion coherence that is suppressed on color context trials. Lines and shaded error bars represent the mean and standard deviation across trials, respectively. **c**, Stimulation of RNN activity along the decoder axis has little effect on the psychometric function. **d**, Stimulation of RNN activity along the motion axis from the latent circuit shifts the decision boundary in the psychometric function consistent with enhanced representation of right motion stimulus. These perturbations reveal that the decoder compromises behavioral relevance for decoding accuracy.

To further test for behavioral relevance of representations identified by different dimensionality reduction methods, we again turned to RNNs in which we can measure behavioral effects of arbitrary activity perturbations. We compared projections of RNN activity onto axes obtained from the latent circuit model and a linear decoder. We trained a linear decoder to predict the signed motion coherence on each trial from RNN activity (Methods). The decoding weights provide an axis in the RNN state space such that a projection onto this axis correlates with the motion coherence. By projecting RNN responses onto the decoder axis, we find a strong representation of irrelevant motion stimulus on color context trials without noticeable suppression (Fig. 7a). Thus, as in the PFC, irrelevant sensory representations in our RNN appear not suppressed along the decoder axis, whereas they appear suppressed along the axis obtained from the latent circuit model (Fig. 7b).

How can we reconcile these qualitatively distinct perspectives on representations of irrelevant stimuli within the same RNN? We hypothesized that the appearance of irrelevant stimulus representations is possible because the linear decoder compromises behavioral relevance for decoding accuracy. To test this idea, we stimulated RNN units with activity patterns aligned with the axes obtained from the linear decoder and the latent circuit model. If the corresponding activity patterns are behaviorally relevant, we expect the stimulation to have a substantial effect on psychometric functions. Specifically, stimulating the representation of the right motion stimulus should increase the proportion of right choices, shifting the decision boundary on motion context trials. As expected, driving RNN activity along the motion axis of the latent circuit model shifted the decision boundary on motion context trials (Fig. 7d). By contrast, stimulation of the same magnitude along the decoder axis had little effect on the psychometric function (Fig. 7c). The irrelevant stimulus representations exist along the decoder axis but do not drive the behavioral output. For context-dependent decision-making, we conclude that the dynamics revealed by the decoder have little behavioral relevance and thus do not invalidate the inhibitory mechanism identified by the latent circuit model. Our results indicate that ‘demixing’ representations of task variables^19,21,42^ may not be the right objective for identifying behaviorally relevant patterns in neural activity and may provide a misleading picture of computation.

## Discussion

Single neurons in higher cortical areas show complex heterogeneous responses during cognitive tasks, posing a challenge for identifying mechanisms of cognitive functions. Our latent circuit model accounts for single-neuron heterogeneity via dimensionality reduction that incorporates low-dimensional circuit dynamics in its latent variables. We show that low-dimensional circuit mechanisms can explain task-relevant dynamics in high-dimensional networks and establish feasibility of inferring these mechanisms from neural response data. Our theory for interpreting the latent circuit connectivity as a low-rank connectivity in RNNs enables causally validating low-dimensional circuit mechanisms via activity and connectivity perturbations in high-dimensional networks. The latent circuit inference can be broadly applied to identify circuit mechanisms for different cognitive tasks from neural response data (Supplementary Fig. 7) and opens new possibilities for causally testing these mechanisms in future experiments.

Although previous studies extensively modeled neural responses using various latent dynamical systems, our work demonstrates feasibility of fitting neural responses with low-dimensional recurrent circuits. Most methods fitting low-dimensional dynamical systems to neural data do not explicitly model task inputs and behavior and do not ground the inferred dynamics in the underlying network connectivity^32,33,40,52,53^. On the other hand, high-dimensional RNN architectures serve as intermediate mechanistic models of neural responses, which generate latent dynamics from high-dimensional recurrent connectivity^25–29^. Although these models can incorporate task inputs and behavioral outputs^27–29,51^, their high-dimensional connectivity is not uniquely constrained by low-dimensional data, limiting possible insights into circuit mechanisms transforming sensory inputs to behavior. Our results motivate future work incorporating low-dimensional recurrent circuits as latent dynamics generators within these model architectures, which will enable combining their ability of fitting single-trial neural activity^26,29^ with the uniqueness and interpretability furnished by the latent circuit model.

By relating connectivity to neural dynamics and behavior, the latent circuit model extends the causal predictive power of classical neural circuit models to the study of mechanisms of cognitive functions in high-dimensional networks. Although dynamical mechanisms have been studied in RNNs by linearizing the RNN flow field around fixed points^19,31^, these dynamical mechanisms do not specify how a particular fixed-point configuration arises from the RNN connectivity. Although dynamical mechanisms can predict changes in activity under perturbations of the dynamical system’s state or inputs, circuit mechanisms provide additional leverage as they can also predict how the dynamical system itself will change under perturbations of activity or connectivity within the network. The causal predictive power of the latent circuit model is further supported by its ability to predict RNN dynamics and behavior for out-of-distribution inputs (Supplementary Fig. 8).

We find that RNNs do not necessarily find qualitatively distinct solutions to cognitive tasks from mechanisms in low-dimensional neural circuit models. We show that low-dimensional mechanisms can be found in large RNNs if connectivity is viewed in the appropriate basis. In other words, just as dynamics can be understood in terms of latent variables^54^, connectivity can be understood in terms of interactions between these latent variables. This perspective is qualitatively similar to previous work on engineering low-dimensional task solutions in RNNs with low-rank connectivity^47,49,55^. However, it was unclear whether low-rank RNNs use mechanisms similar to classical circuit models or implement truly novel solutions that emerge only in high-dimensional nonlinear systems. Our work explicitly relates low-dimensional recurrent circuits and low-rank connectivity in RNNs and enables inferring latent circuit structure in generic RNNs trained without low-rank connectivity constraints. We find that these generic RNNs also use low-rank connectivity to perform the task, although their full high-dimensional connectivity is not necessarily low rank.

We found that RNNs trained on a context-dependent decision-making task use a suppression mechanism in which context nodes inhibit irrelevant sensory nodes. The inhibitory suppression mechanism revealed by the latent circuit model is qualitatively similar to previous neural circuit models of how PFC flexibly switches between alternative stimulus–response mappings^8–10,44,45^. Thus, RNNs do not find qualitatively distinct solutions to this task, and complex selectivity of single neurons has a simple explanation as a linear mixing of the low-dimensional latent inhibitory circuit mechanism. Although we found a variety of task solutions across RNNs trained with the same hyperparameters to the same performance level, all these solutions were based on the same suppression mechanism. In general, a solution to which an RNN converges may depend on numerous hyperparameters (for example, for initialization and regularization), and the latent circuit inference offers a quantitative tool for characterizing how these hyperparameters influence the space of task solutions.

The latent circuit model revealed a suppression of irrelevant sensory representations in PFC responses of monkeys performing a context-dependent decision-making task. By contrast, correlation-based dimensionality reduction methods found no significant suppression of irrelevant stimuli in the same data^19,21^. This difference results from recurrent interactions among task variables in the latent circuit model, and dimensionality reduction methods that do not incorporate these interactions are biased toward uncovering sensory representations that are not modulated by context. Considering interactions among task variables is crucial as they implement the computations necessary to solve the task, and omitting them provides a misleading picture of computation. We show that representations of irrelevant stimuli also exist in RNNs that provably implement an inhibitory suppression mechanism, but these representations do not causally drive choices. Thus, inhibitory mechanisms for cognitive flexibility are compatible with the existence of irrelevant stimulus representations in the PFC.

The latent circuit model opens a route for interpreting circuit mechanisms in high-dimensional networks. A prerequisite for a model to be interpretable is its uniqueness^40^. Whereas low-dimensional data do not uniquely constrain the full high-dimensional connectivity in RNNs, we show that we can uniquely recover the latent low-rank connectivity within a high-dimensional network, which therefore can be reliably interpreted. We operationally define interpretation of the circuit as the ability to achieve prescribed behavioral effects through perturbations of specific nodes or connections (Figs. 4 and 7). In general, small recurrent circuits can generate complex dynamics that are difficult to intuit from connectivity alone^56^. In such cases, we can dissect the circuit function by analyzing the effects of connectivity perturbations on the dynamics and behavior^57^. Our work translates such circuit dissection methods^56,57^ to high-dimensional networks in which, unlike in small circuits, individual connections do not carry specific functions, but instead these functions arise from distributed connectivity patterns.

Interpreting circuit mechanisms is more accessible in low-dimensional tasks, which have been extensively used to probe functions of higher cortical areas. Cumulative findings suggest that similar dynamical and circuit motifs are used across many tasks, and, moreover, solutions to more complex tasks can arise by composing simple motifs^1,24,58^. Consistent with this idea, we find that RNNs trained on different tasks use a similar inhibitory control mechanism (Supplementary Fig. 7), which may generalize to other cognitive control tasks as well. Therefore, low-dimensional circuit mechanisms may provide the primitives for building more complex cognitive functions, and our work suggests a path forward for interpreting these mechanisms in neural data.

## Methods

### Fitting a latent circuit model

We fit the latent circuit model Eqs. (1)–(3) to neural response data *y* by minimizing the mean squared error loss function,

7 $$L=\sum _{k}\sum _{t}\parallel {y}_{tk}-Q{x}_{tk}{\parallel }_{2}+\parallel {z}_{tk}-{w}_{{\rm{out}}}{x}_{tk}{\parallel }_{2},$$

using custom Python code^59^. Here, *k* indexes the trials, *t* indexes the time within a trial, and *Q* is an orthonormal embedding matrix. Because the variable *x* depends implicitly on the latent circuit parameters *w*_rec_ and *w*_in_, the minimization of *L* is a nonlinear least squares optimization problem^60^ in which we simultaneously search for a behaviorally relevant projection of the high-dimensional activity and a low-dimensional neural circuit that generates dynamics in this projection. Because orthonormal matrices define a nonlinear submanifold within the space of all matrices, minimizing *L* corresponds to solving a constrained optimization problem over this submanifold. To transform it into an unconstrained problem, we use the Cayley transform to parameterize orthonormal matrices by the linear space of skew symmetric matrices^61^,

8 $$Q=(I+A){(I-A)}^{-1}{\pi }_{n},$$

where *π*_*n*_ represents projection onto the first *n* columns, and *A* is skew symmetric. We parameterize *A* by an arbitrary square *N* × *N* matrix *B*,

9 $$A=B-{B}^{T}.$$

With these reparameterizations, we can minimize *L* over the vector space of square matrices *B*. The parameterization of a skew symmetric matrix *A* with the auxiliary matrix *B* has a degeneracy because *A* has only *N*(*N* − 1) / 2 distinct elements. We did not attempt to eliminate this degeneracy because *B* is an auxiliary matrix, and we did not observe any degeneracy arising in matrix *Q* during fitting.

At each step of the optimization, we generate a set of trajectories *x* from the latent circuit dynamics and embed these trajectories using the matrix *Q*. The parameters *B*, *w*_rec_, *w*_in_ and *w*_out_ are then updated to minimize *L*. We perform this minimization using PyTorch and the Adam optimizer with default values 0.9 and 0.999 for the decay rate of the first and second moment estimates, respectively, a learning rate of 0.02 and a weight decay of 0.001. We use a minibatch size of 128 trials. We stop the optimization when the loss has not improved by a threshold of 0.001 after a patience of 25 epochs. We used the Python software package Seaborn for visualizing model parameters and responses after training.

We initialize the recurrent matrix *w*_rec_ from a uniform distribution centered on 0 with a standard deviation of 1/*n*. We initialize *w*_in_ with zeros except for positive entries along the diagonal on connections from inputs *u* to their corresponding nodes and *w*_out_ with zeros except for positive entries on connections from choice nodes to their corresponding outputs *z*. We initialize the entries of matrix *B* from a uniform distribution on [0, 1].

When fitting the latent circuit model, we found some amount of variability in solutions across multiple optimization runs with different initialization. To control for this variability, we fitted a large ensemble of latent circuit models (*n* = 100) with different initialization of the parameters for latent connectivity and embedding *Q* for each RNN model. This ensemble of latent circuit models for a single RNN has variable fit quality because many optimization runs do not converge to the optimal solution (which is typical for nonconvex optimization). Therefore, we selected the best ten latent circuit models from this ensemble in terms of fit quality on held out test data, which formed a set of converged solutions (Fig. 5a). To quantify the uniqueness of the latent circuit solution in each RNN, we computed the correlation coefficients between the recurrent connectivity weights of the best model and the remaining nine converged models. We can use the correlation coefficient because the identity of each node in the latent circuit is defined by its input and output connectivity, eliminating any permutation symmetries.

### Testing the dependence of latent connectivity on neural responses

To determine whether the inferred latent circuit connectivity significantly depends on neural response data beyond the constraints imposed by the task alone, we performed a permutation test (Extended Data Figs. 1, 7c and 8c), which proceeds in three steps. First, we fit *N* latent circuit models to neural responses and select the best model in terms of fit quality on held out test data. We then compute the correlation coefficients between recurrent connectivity of the best model and all other models. The distribution of these correlation coefficients estimates how variable the latent connectivity is across models fitted to the original neural responses. Second, we shuffle neural responses *N* times and fit a latent circuit model to each shuffle, resulting in *N* latent circuit models. Our shuffling procedure randomly permutes neural responses with respect to trial conditions while preserving the input–output relationship on each trial so that the fitted latent circuit models can still perform the task. We confirm that the latent circuit models fitted to the shuffled RNN responses perform the task at high accuracy (Extended Data Fig. 1). These latent models serve as a control to assess whether the inferred latent connectivity emerges merely from the task constraints alone, and they should not be viewed as models of any specific high-dimensional network. We then compute the correlation coefficients between the connectivity of all models fitted to the shuffled data and the best model from the original data fit. Third, we use a Mann–Whitney *U*-test to determine whether the correlation coefficients are significantly smaller for models fitted to the shuffled responses than original neural responses. This outcome would indicate that models fitted to shuffled neural responses use more diverse connectivity to perform the task than models fitted to the original data; thus, neural responses significantly constrain the inferred connectivity above the effects of the task. We used the same test for both RNN (*N* = 500; Extended Data Fig. 1) and PFC data (*N* = 1,000; Extended Data Figs. 7c and 8c).

### Relationship between connectivity of the RNN and latent circuit

We consider RNNs of the form

10 $$\tau \dot{y}=-y+{\left[{W}_{{\rm{rec}}}\,y+{W}_{{\rm{in}}}u\right]}_{+}.$$

Here, [⋅]_+_ is a rectified linear (ReLU) activation function, *τ* is a time constant, and *u* are external task inputs. *W*_rec_ and *W*_in_ are the recurrent and input connectivity matrices, respectively. We read out a set of task outputs *z* from the network activity via the output connectivity matrix *W*_out_,

11 $$z={W}_{{\rm{out}}}\,y.$$

We derive a relationship between the connectivity matrices of the RNN and latent circuit, which allows us to interpret the latent circuit connectivity as a latent connectivity structure in the RNN. To derive this relationship, we differentiate the embedding Eq. (1) with respect to time and obtain the relationship between the vector fields of the RNN and latent circuit,

$$V(\,y)=Qv(x).$$

Here, the vector fields $\dot{y}=V(\,y)$ of the RNN and $\dot{x}=v(x)$ of the latent circuit are given by Eqs. (10) and (2), respectively. This equation states that the subspace spanned by the columns of *Q* is an invariant subspace of the high-dimensional system; that is, the vector field at any point in this subspace lies entirely in this subspace. We then use the orthonormality condition *Q*^*T*^*Q* = *I* to obtain

$${Q}^{T}V(Qx)=v(x).$$

Substituting the vector fields Eq. (10) and Eq. (2) in this relation gives us the equality

12 $${Q}^{T}{[{W}_{{\rm{rec}}}Qx+{W}_{{\rm{in}}}u]}_{+}={[{w}_{{\rm{rec}}}x+{w}_{{\rm{in}}}u]}_{+}.$$

Because this is an equality of two piecewise-linear systems, it holds for each local linear piece individually. In particular, assuming that both the inputs *u* and *W*_in_ are positive, we take *x* sufficiently near 0, where the argument of the nonlinearity is positive for all units. In this local linear piece, we have the equality of linear systems

13 $${Q}^{T}{W}_{{\rm{rec}}}Qx+{Q}^{T}{W}_{{\rm{in}}}u={w}_{{\rm{rec}}}x+{w}_{{\rm{in}}}u.$$

If we assume that this equality holds in some open set, then we can equate the terms to obtain an equality of connectivity matrices:

14 $${Q}^{T}{W}_{{\rm{rec}}}Q={w}_{{\rm{rec}}},$$

15 $${Q}^{T}{W}_{{\rm{in}}}={w}_{{\rm{in}}}.$$

This assumption is likely not fully satisfied in the setting of cognitive tasks because the sets of inputs *u* and latent states *x* are typically low dimensional. Therefore, the above equalities may hold only approximately. In addition, the equality of piecewise-linear dynamical systems Eq. (12) depends on the correspondence between trajectories of the RNN and latent circuit Eq. (1). Because, in practice, we search for the latent circuit by minimizing the loss function *L*, if *L* is not exactly equal to 0, then Eq. (1) and consequently Eqs. (14) and (15) hold only approximately.

We derived the analytical relations between connectivity in the latent circuit and RNN (Eqs. (14) and (15)) assuming that the latent circuit provides a good fit of RNN responses and that their dynamical equations (Eqs. (2) and (10)) have the same nonlinearity. In general, it is unclear whether a latent circuit model can satisfactorily fit responses of a high-dimensional network that has a different nonlinearity and to what extent the relation between their connectivity will hold in this case. To test whether our results extend to networks with a different biologically plausible nonlinearity, we trained RNNs that had a Softplus activation function $f(x)=\frac{g}{\beta }\log (1+{e}^{\beta x})$ for a range of parameter *β* and also with varying gain *g* across units. We fitted responses of these RNNs with our latent circuit model that had a rectified linear (ReLU) activation function and found that this architecture mismatch did not significantly affect the fit quality and the relationship between connectivity (Extended Data Figs. 5 and 6).

To understand how perturbations of connectivity in the latent circuit map onto the RNN, we view perturbations as vectors in the space of matrices. We denote *A* ⋅ *B* the dot product between the matrices *A* and *B* represented as vectors in the space of matrices; that is, *A* ⋅ *B* = ∑_*i*_∑_*j*_*A*_*ij*_*B*_*ij*_. Using Eqs. (14) and (15), we then translate connectivity perturbations from the latent circuit to the RNN:

16 $${w}_{ji}=w\cdot {\delta }_{ji}={({Q}^{T}WQ)}_{ji}$$

17 $$=\mathop{\sum }\limits_{k=1}^{N}{Q}_{kj}\left(\mathop{\sum }\limits_{l=1}^{N}{W}_{kl}{Q}_{li}\right)$$

18 $$=\mathop{\sum }\limits_{k=1}^{N}\mathop{\sum }\limits_{l=1}^{N}{W}_{kl}{Q}_{kj}{Q}_{li}$$

19 $$=\mathop{\sum }\limits_{k=1}^{N}\mathop{\sum }\limits_{l=1}^{N}{W}_{kl}{({q}_{j}{q}_{i}^{T})}_{kl}$$

20 $$=W\cdot {q}_{j}{q}_{i}^{T}$$

21 $$=W\cdot Q{e}_{j}{(Q{e}_{i})}^{T}$$

22 $$=W\cdot Q{\delta }_{ji}{Q}^{T},$$

where *q*_*i*_ is the *i*th column of *Q*, and *e*_*i*_ is the *i*th standard unit vector. This chain of equalities shows how to translate perturbations of the latent circuit connectivity in the direction *δ*_*ji*_ onto rank-one connectivity perturbations in the RNN,

23 $$w\cdot {\delta }_{ji}=W\cdot Q{\delta }_{ji}{Q}^{T}.$$

Thus, to perturb the latent connection *w*_*ji*_, we perturb the matrix *W* in the direction *Qδ*_*ji*_*Q*^*T*^. In other words, to increase the dot product between *W* and *Qδ*_*ji*_*Q*^*T*^ in the space of matrices, we add multiples of *Qδ*_*ji*_*Q*^*T*^ to *W*. Any perturbation orthogonal to *Qδ*_*ji*_*Q*^*T*^ does not change the dot product and hence has no effect on the latent connection *w*_*ji*_.

### RNN simulations

We simulate dynamics of time-discretized RNNs using the general framework for modeling cognitive tasks^22^. We consider RNNs with positive activity and *N* = 50 recurrent units. We obtained the same results with networks consisting of *N* = 150 units. We discretize the RNN dynamics Eq. (10) using the first-order Euler scheme with a time step Δ*t* and add a noise term to obtain

24 $${y}_{t}=(1-\alpha ){y}_{t-1}+\alpha {\left[{W}_{{\rm{rec}}}{\,y}_{t-1}+{W}_{{\rm{in}}}{u}_{t}+\sqrt{\frac{2}{\alpha }}{\sigma }_{{\rm{rec}}}{\xi }_{t}\right]}_{+}.$$

Here, *α* = Δ*t*/*τ* and ${\xi }_{t} \sim {\mathcal{N}}(0,1)$ is a random variable sampled from the standard normal distribution. We set the time constant *τ* = 200 ms, the discretization time step Δ*t* = 40 ms, and the noise magnitude *σ*_rec_ = 0.15. When fitting RNN responses with the latent circuit model, we discretize the latent circuit dynamics Eq. (2) using the same hyperparameter *α* and the same noise magnitude as was used when training the RNN. The input and output matrices are constrained to have positive entries. The recurrent matrix is constrained to satisfy Dale’s law with 80% excitatory units and 20% inhibitory units. For RNNs shown in the main text, the concatenation of input and output matrices is constrained to be orthogonal. However, our conclusions do not depend on this constraint, and we find similar latent circuit fits and the inhibitory mechanism in RNNs trained with unconstrained inputs (Supplementary Fig. 6). The RNN simulation and training were implemented in Python using the software package PyTorch.

### Context-dependent decision-making task

In the context-dependent decision-making task, at the beginning of each trial, a context cue briefly appears to indicate either the color or motion context for the current trial. After a short delay, a sensory stimulus appears that consists of motion and color features. The right motion and red color are associated with the right choice, and the left motion and green color are associated with the left choice. The strength of motion and color stimuli varies from trial to trial as quantified by the motion and color coherence. In the color context, the choice should be made according to the color, ignoring the motion stimulus, and vice versa in the motion context.

To model the context-dependent decision-making task, the network receives six inputs *u* corresponding to two context cues (*u*_m_: motion context; *u*_c_: color context) and sensory evidence streams for motion (*u*_m,L_: motion left; *u*_m,R_: motion right) and color (*u*_c,R_: color red; *u*_c,G_: color green). The network has two outputs, *z*_1_ and *z*_2_, for which we define two targets *z*_target,1_ and *z*_target,2_. Each trial begins with a presentation of a context cue from *t* = 320 to *t* = 1, 000 ms. On motion context trials, the cue input is set to *u*_m_ = 1.2 and *u*_c_ = 0.2 and vice versa on color context trials. During this epoch, we require that the network does not respond on the outputs by setting *z*_target_ = 0.2. After a delay of 200 ms, so that the network must maintain a memory of the context cue, the inputs corresponding to motion and color sensory evidence are presented at *t* = 1,200 ms for the remaining duration of the trial. From 2,250 ms after the start of the trial and extending to the end of the trial, the targets are defined by *z*_target,1_ = 1.2 and *z*_target,2_ = 0.2 for right choices and vice versa for left choices. The strength of sensory evidence for motion and color varies randomly from trial to trial controlled by the stimulus coherence. We use motion coherence *m*_c_ and color coherence *c*_c_ ranging from −0.2 to 0.2 chosen from the set {−0.2, −0.12, −0.04, 0.04, 0.12, 0.2}. For each coherence level, the motion and color inputs are given by

$$\begin{array}{rcl}{u}_{{\rm{m}},{\rm{L}}}=\frac{1-{m}_{{\rm{c}}}}{2},&&\;\;{u}_{{\rm{m}},{\rm{R}}}=\frac{1+{m}_{{\rm{c}}}}{2},\\ {u}_{{\rm{c}},{\rm{G}}}=\frac{1-{c}_{{\rm{c}}}}{2},&&\;\;{u}_{{\rm{c}},{\rm{R}}}=\frac{1+{c}_{{\rm{c}}}}{2}.\end{array}$$

With these definitions, positive motion and color coherence provide evidence for the right choice, and negative motion and color coherence provide evidence for the left choice. At each simulation time step, we add an independent noise term to each of the inputs ${u}_{{\rm{noise}}}=\sqrt{2{\alpha }^{-1}}{\sigma }_{{\rm{in}}}{\eta }_{t}$, where ${\eta }_{t} \sim {\mathcal{N}}(0,1)$ is a random variable sampled from the standard normal distribution. The input noise strength is *σ*_in_ = 0.01. A baseline input *u*_0_ = 0.2 is added to each of the inputs at each time step.

### RNN training

To train the RNN, we minimize the mean squared error between the output *z*(*t*) and the target *z*_target_(*t*):

25 $${\mathcal{L}}:= \sum _{ikt}{({z}_{ikt}-{z}_{{\rm{target}},ikt})}^{2}+{\lambda }_{r}\sum _{ikt}{y}_{ikt}^{2}.$$

Here, *k* is the trial number, *t* is the time step within a trial, *z*_*ikt*_ is the *i*th output on trial *k* and time *t*, and *y*_*ikt*_ is the response of the *i*th RNN unit on trial *k* at time *t*. The first term is the task error, and the second term serves to regularize by penalizing the magnitude of the firing rates. To encourage the network to integrate sensory evidence over time and to not output responses during the context cue, these task errors are only penalized in the last 750 ms of each trial and during the presentation of the contextual cue. The training is performed with the Adam algorithm. We used the default values 0.9 and 0.999 for the decay rate of the first and second moment estimates, respectively. We used a learning rate of 0.01 and a weight decay of 0.001 and set the hyperparameter *λ*_*r*_ = 0.05.

We control the degree of correlation between the input and output vectors in the RNN by adding an *L*_2_ penalty

26 $${\lambda }_{{\rm{orth}}}\parallel {B}^{T}B-\,\text{diag}\,({B}^{T}B){\parallel }_{2}$$

to the loss function in Eq. (25) during training. Here, *B* is the matrix corresponding to the concatenation of *W*_in_ and ${W}_{{\rm{out}}}^{T}$ along their second dimension, with columns normalized to unit length. The hyperparameter *λ*_orth_ controls the penalty weight. For RNNs in the main text, we set *λ*_orth_ = 1, which results in nearly orthogonal input vectors (Supplementary Fig. 6). We fit responses of these RNNs with latent circuit models in which the matrix *B* is constrained to be diagonal during fitting by setting off-diagonal elements to 0 after each gradient update. By setting *λ*_orth_ to a smaller value during RNN training, the input vectors in the trained RNN become slightly correlated (Supplementary Fig. 6). To test the effect of these correlations in the latent circuit model, we add the penalty Eq. (26) to the loss function Eq. (7) during latent circuit fitting (Supplementary Fig. 6). These correlations can be captured in the latent circuit model fitted with smaller values of the corresponding *λ*_orth_ hyperparameter. Allowing for these input correlations in RNNs and the latent circuit does not have a strong effect on either fits or the underlying circuit mechanism (Supplementary Fig. 6).

The recurrent connection matrix *W*_rec_ is initialized so that excitatory connections are independent Gaussian random variables with mean $1/\sqrt{N}$ and variance 1/*N*. Inhibitory connections are initialized with mean $4/\sqrt{N}$ and variance 1/*N*. The matrix is then scaled so that its spectral radius is 1.5. To implement Dale’s law, connections are clipped to 0 after each training step if they change sign. During training, we used minibatches of 128 trials with 1,800 trials total.

To assess performance, a choice for the RNN was defined as the sign of the difference between output units at the end of the trial. Psychometric functions were then computed as the percentage of choices to the right for each combination of context, motion coherence and color coherence.

### Linear decoding

To decode motion coherence from RNN responses, we fit a linear regression model

27 $$c=\beta y+b,$$

where $\beta \in {{\mathbb{R}}}^{1\times N}$ is the vector of regression coefficients, $c\in {{\mathbb{R}}}^{1\times K\cdot T}$ is the motion coherence on each trial, $b\in {\mathbb{R}}$ is a bias term, and $y\in {{\mathbb{R}}}^{N\times K\cdot T}$ is the RNN responses at each time step during the stimulus epoch of each trial. Here, *K* is the number of trials, and *T* is the number of time points within a trial. We split the data into training and test sets and fit the model on the training set. There was no large difference between training and test scores (*r*^2^ = 0.535 and *r*^2^ = 0.531), suggesting that the model did not overfit. After fitting, we used the vector of regression coefficients *β* to define the decoder axis on which we project RNN responses.

### Analysis of PFC data

We analyzed a publicly available dataset of neural activity recordings from the PFC (in and around the frontal eye field) from two monkeys performing a context-dependent decision-making task^19^. This dataset consisted of 762 units from monkey A and 640 units from monkey F (including single neurons and multiunits). To facilitate comparison with previous studies analyzing the same dataset^19,21^, we used identical initial preprocessing of the neural data (using the publicly available code at https://www.ini.uzh.ch/en/research/groups/mante/data.html). Because stimulus coherence levels varied across monkeys and days, to equate performance in the motion and color contexts, we replaced the coherences on each trial with their average values for each stimulus difficulty (average motion coherences: 0.05, 0.15 and 0.50 in monkey A and 0.07, 0.19 and 0.54 in monkey F; average color coherences: 0.06, 0.18 and 0.50 in monkey A and 0.12, 0.30 and 0.75 in monkey F). Monkeys reported their choice with a saccade to one of two targets presented shortly after fixation for the entire trial duration. The monkeys were rewarded for saccades to the target location corresponding to the motion direction in the motion context and to the target whose color matched the dominant color of the dots in the color context. The stimulus coherence was assigned a sign (positive or negative) according to the target location indicated by the stimulus. Because the color of the targets was randomized between locations on each trial, the sign of the color coherence reflects both the dominant color of the dots and the location of the red and green targets. The task therefore had 72 unique stimulus conditions defined by all combinations of six motion coherence levels, six color coherence levels and two contexts.

We fitted the latent circuit model to trial-averaged neural responses on correct trials. In our analyses, we included neurons that had at least four correct trials for each of the 72 unique trial conditions, which produced 483 neurons for monkey A and 323 neurons for monkey F. For cross-validation, we then split the trials into two equal disjoint sets and computed the trial-averaged response of each neuron for each trial condition within each set. We used the training set for model fitting and the validation set for visualizing projections of neural responses and quantifying the fit quality. For the analysis of error trials (Extended Data Figs. 7d and 8d), we considered the set of error trial conditions for which all analyzed neurons had at least one trial, which resulted in 16 conditions for monkey A and 26 conditions for monkey F. We then computed the trial-averaged response of each neuron for each trial condition within this set of error trials.

We analyzed neural responses during the presentation of the random dots stimulus because the available data consisted of neural responses starting at 100 ms after stimulus onset for a duration of 750 ms. For each trial, we computed time-varying firing rates by counting spikes in a 50-ms sliding square window (50-ms steps). The first window was centered at 100 ms after the onset of the stimulus, and the last window was centered at 100 ms after stimulus offset. Within the training and test sets, we *z* scored and smoothed (Gaussian kernel, *σ* = 40 ms) the response of each unit. Following previous studies^19^, from activity of each unit we subtracted a condition-independent term corresponding to the mean response at each time across trial conditions. To construct population responses, we combined the single-neuron responses for each trial condition. This resulted in 72 neural trajectories for each combination of context, motion coherence and color coherence. Last, to denoise these trajectories, we projected them onto the principal components explaining 50% of their total variance (corresponding to the first 40 and 31 principal components for monkeys A and F, respectively).

We fitted latent circuit models to the PFC data following similar procedures as for RNNs. For each of the 72 conditions, we constructed input to the latent circuit from the context, motion and color coherence corresponding to that condition. In the experimental task, the stimulus is presented 650 ms after the context cue for a duration of 750 ms. Neural recordings correspond to 100 ms after stimulus onset to 100 ms after stimulus offset. We thus modeled the task with 150 time steps (10 ms in length) extending from the initial presentation of the contextual cue to 100 ms after stimulus offset. Contextual input was given to the model from *t* = 0 to *t* = 1,500 ms. Stimulus input was given to the model from *t* = 750 ms to *t* = 1,500 ms. We constructed two target outputs (*z*_1_ and *z*_2_) for each trial such that on trials for which the monkey chose the right target, the first target output was high (*z*_1_ = 1.2) and the second target output was low (*z*_2_ = 0.2) and vice versa for the left choice trials. We penalized errors between target and model outputs only in the last 250 ms of each trial. Responses of the latent circuit were fitted to the PFC data only on the last 15 time steps of each trajectory for which there were available PFC data. The latent circuit model was fitted with hyperparameter *α* = 0.2. The latent circuit model was fitted with a recurrent noise term of magnitude *σ*_rec_ = 0.15, which was added to each unit at each time step (Eq. (24)). Because neural responses were centered, we additionally fit an intercept term *b* so that the resulting model for PFC data was

28 $$y=Qx+b,$$

29 $$\tau \dot{x}=-x+{[{w}_{{\rm{rec}}}x+{w}_{{\rm{in}}}u]}_{+},\,{\rm{and}}$$

30 $$z={w}_{{\rm{out}}}x.$$

Because of high dimensionality of PFC responses (40 and 31 principal components are required to account for ~50% of the total variance in PFC activity for monkeys A and F, respectively), we find a notable tradeoff between the task fit and data fit when fitting the low-dimensional latent circuit model to the PFC data. To control this tradeoff, we used a modified loss function when fitting PFC data,

31 $$L=\sum _{k}\sum _{t}\lambda \frac{\parallel y-Q{Q}^{T}y{\parallel }_{2}}{\parallel y{\parallel }_{2}}+\frac{\parallel {Q}^{T}(\,y-b)-x{\parallel }_{2}}{\parallel {Q}^{T}y{\parallel }_{2}}+\frac{\parallel z-{w}_{{\rm{out}}}x{\parallel }_{2}}{\parallel z{\parallel }_{2}}$$

32 $$=\lambda {r}_{Q}^{2}+{r}_{x}^{2}+{r}_{z}^{2},$$

designed to balance variance explained by the task-relevant subspace ${r}_{Q}^{2}$, the fits between projected PFC responses and the latent circuit trajectories in this subspace ${r}_{x}^{2}$ and the performance of the latent circuit on the task ${r}_{z}^{2}$. The hyperparameter *λ* = 0.5 was chosen via a grid search over the range *λ* ∈ [0, 1.5]. We found that near this value, the metrics ${r}_{x}^{2}$ and ${r}_{Q}^{2}$ were maximized under the constraint that the latent model still performed well on the task (Extended Data Figs. 7a and 8a).

### Statistics and reproducibility

We analyzed data from 200 RNN models trained with random initializations. Results were consistent across networks; therefore, we found this sample size to be sufficient for our study. No statistical method was used to predetermine sample size. For each of these networks, we trained 100 latent circuit models. This sample size was chosen so that the top ten latent models converged to a high fit accuracy. For PFC data, we fitted 200 latent circuit models to neural responses from each monkey. Neural recording data were previously described in Mante et al.^19^; no randomization or blinding was performed because there was only one experimental group. All recorded units that had at least four correct trials in each task condition were included in the analysis.

### Reporting summary

Further information on research design is available in the Nature Portfolio Reporting Summary linked to this article.

## Online content

Any methods, additional references, Nature Portfolio reporting summaries, source data, extended data, supplementary information, acknowledgements, peer review information; details of author contributions and competing interests; and statements of data and code availability are available at 10.1038/s41593-025-01869-7.

## Supplementary information

Supplementary Information Supplementary Figs. 1–8.

Reporting Summary

## Source data

Source Data Fig. 5 Connectivity parameters and training hyperparameters for 200 RNNs trained on a context-dependent decision-making task. Connectivity parameters and training hyperparameters for latent circuit models fit to RNNs.

Source Data Fig. 6 Parameters for latent circuit models fit to monkey A PFC data.

Source Data Extended Data Fig. 9 Parameters for latent circuit models fit to monkey F PFC data.

## Data Availability

Synthetic data used in this study can be reproduced using the source code with model parameters included in the Source Data files provided with this paper. Neural recording data were previously described in Mante et al.^19^ and are available in a public repository at https://www.ini.uzh.ch/en/research/groups/mante/data.html. Source data are provided with this paper.

## Code availability

The source code to reproduce the results of this study is publicly available on GitHub (https://github.com/engellab/latentcircuit) and Zenodo (https://zenodo.org/records/14020108)^59^.
