# Supplementary material for: Latent circuit inference from heterogeneous neural responses during cognitive tasks
Source: Nat Neurosci. 2025 Feb 10;28(3):665–75. doi: 10.1038/s41593-025-01869-7 (PMC11893458; doi:10.1038/s41593-025-01869-7)
Supplement: Supplementary file 1 — Supplementary Figs. 1–8. [file 41593_2025_1869_MOESM1_ESM.pdf]

nature neuroscience

Supplementary information

https://doi.org/10.1038/s41593-025-01869-7

Latent circuit inference from heterogeneous neural responses during cognitive tasks

In the format provided by the authors and unedited

Supplementary Information for: Latent circuit inference from heterogeneoous neural responses during cognitive tasks

Christopher Langdon and Tatiana A. Engel

Princeton Neuroscience Institute, Princeton University, Princeton, NJ 08540

Cold Spring Harbor Laboratory, Cold Spring Harbor, NY 11724

Corresponding author e-mail: tatiana.engel@princeton.edu

November 2, 2024

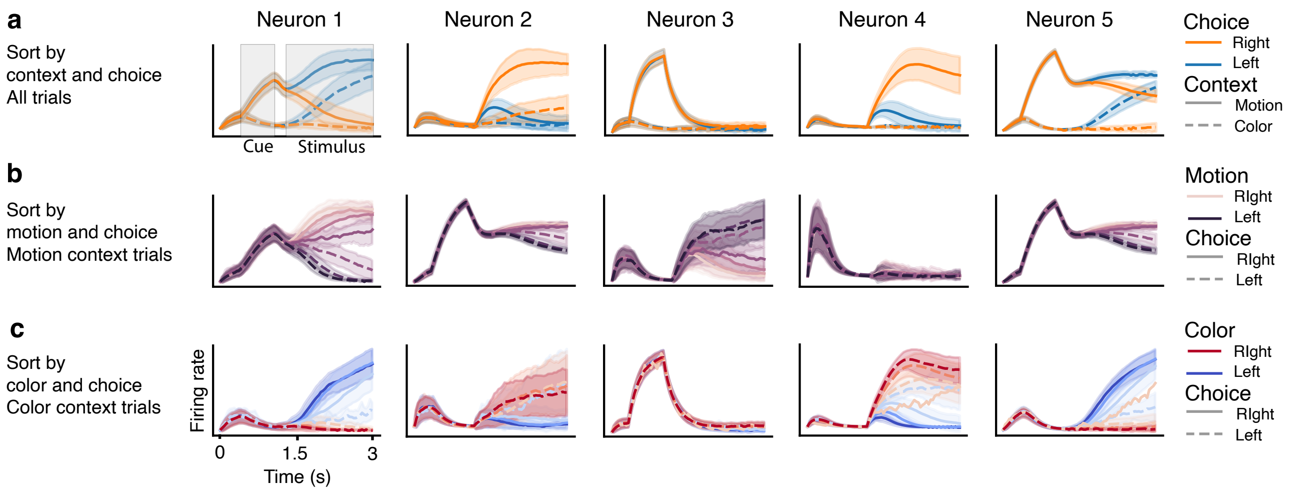

Supplementary Figure 1. Heterogeneous responses of single units in task-optimized RNNs. Responses of five example units (columns) from an RNN model of context-dependent decision-making task. Responses are aligned to trial start and sorted by one or more task variables. Gray shading indicates the context (Cue) and stimulus epochs (Stimulus). (**a**) Responses are sorted by context and choice. Different neurons are selective for context, choice or both. (**b**) Responses are sorted by motion coherence and choice, restricted to motion context trials. Different neurons are selective for both motion coherence and choice, or neither. (**c**) Responses are sorted by color coherence and choice, restricted to color context trials. Different neurons are selective for color coherence, choice, neither or both. In all panels, lines and error bars represent the mean and one standard deviation computed across trials.

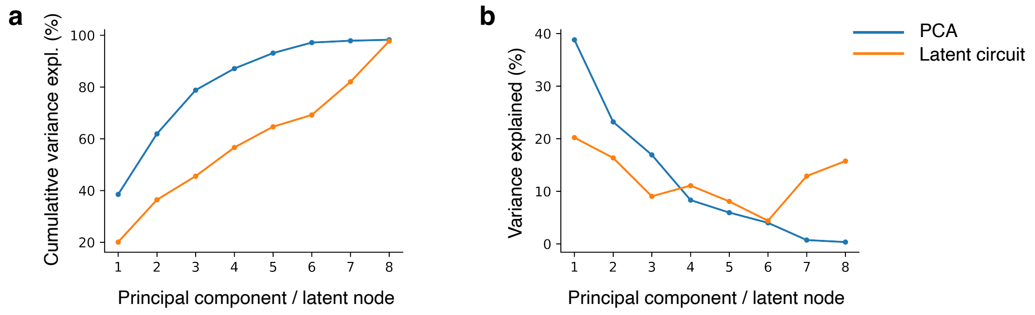

Supplementary Figure 2. Comparison of the explained variance in RNN responses by principal component analysis (PCA) and latent circuit model. (**a**) Cumulative explained variance in RNN responses by the first eight principal components (PCs, blue) and the eight columns of the embedding matrix Q from the latent circuit model (orange). Both the first eight PCs and the latent circuit model capture nearly all variance in the RNN responses. Thus, the embedding matrix of the latent circuit model can be viewed as an alternative basis for the subspace spanned by the first eight PCs. (**b**) Percentage of explained variance in RNN responses by the first eight PCs (blue) and the columns of the embedding matrix Q from the latent circuit model (orange). In contrast to PCA, the activity of nodes in the latent circuit model is not aligned with the directions of maximal variance, but instead the basis provided by the latent nodes captures interactions among task variables. Data are for the RNN and latent circuit model from Fig. 3 in the main text.

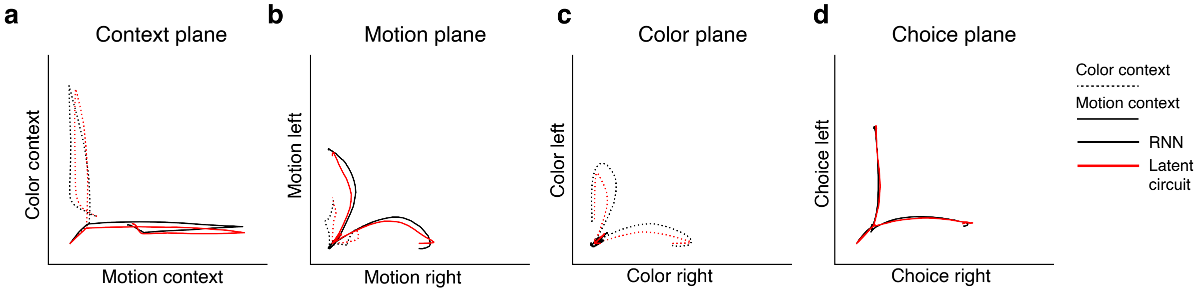

Supplementary Figure 3. Agreement between the latent circuit and projected RNN trajectories. The latent circuit dynamics capture the projection of RNN responses onto 8-dimensional subspace spanned by columns of the embedding matrix Q. The latent circuit trajectories (red) closely match the projected RNN responses (black). (**a**) Projection onto two-dimensional subspace spanned by motion-context and color-context axes. Two trajectories show the mean response on motion context (solid line) and color context (dashed line) trials. (**b**) Projection onto two-dimensional subspace spanned by motion-right and motion-left axes. Four trajectories show the mean response for all positive motion coherences and all negative motion coherences on motion context (solid line) and color context (dashed line) trials. The projection reveals that activity in the motion-subspace is suppressed toward the origin on color-context trials. (**c**) Projection onto two-dimensional subspace spanned by color-right and color-left axes. Trajectories correspond to the mean response for all positive color coherences and all negative color coherences on motion context (solid line) and color context (dashed line) trials. The projection reveals that activity in the color-subspace is suppressed toward the origin on motion context trials. (**d**) Projection onto two-dimensional subspace spanned by choice-right and choice-left axes.

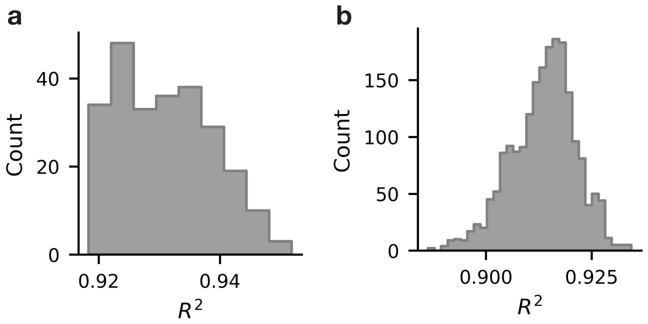

Supplementary Figure 4. Task performance and latent circuit fits for an ensemble of 200 RNNs. (**a**) Histogram of performance metric across 200 RNNs trained on the context-dependent decision-making task. The performance metric is the coefficient of determination (R^2) between RNN output and targets on test data. All RNNs achieve similar task performance. (**b**) Histogram of latent circuit fit quality for the RNN ensemble in a. Fit quality is measured by coefficient of determination (R^2) between RNN responses and embedded latent circuit responses on test data. For each RNN, we perform 100 latent circuit fits starting with random initializations. Distribution shows top 10 fits for each RNN. All latent circuits accurately fit RNN responses.

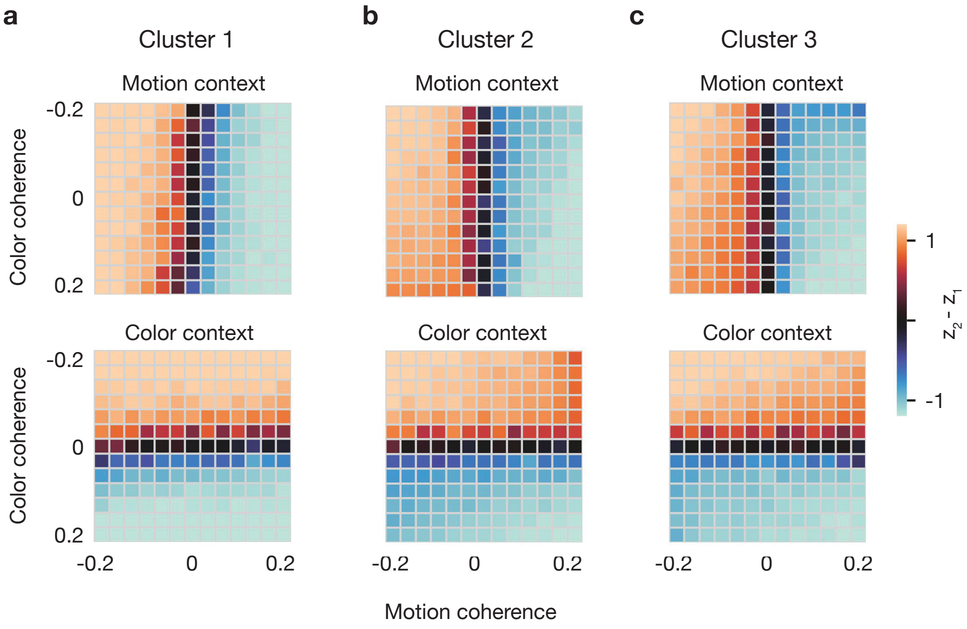

Supplementary Figure 5. Consistency of task performance across RNNs with different latent circuit structure. In the ensemble of 200 RNNs trained on the context-dependent decision-making task, latent circuit solutions form three major clusters (Fig. 5 in the main text). RNNs in different clusters differ in the latent connectivity structure and dynamics, but they all show consistent task performance. (**a**) Mean difference between two output units at the trial end for each task condition, averaged over all RNNs in cluster 1. Cluster 1 corresponds to the symmetric inhibitory mechanism. (**b**) Same as a for RNNs in cluster 2, which corresponds to an asymmetric inhibitory mechanism. (**c**) Same as a for RNNs in cluster 3, which corresponds to another asymmetric inhibitory mechanism. The consistency of RNN outputs in different clusters indicates that differences in latent circuit mechanisms between clusters do not arise from differences in task performance.

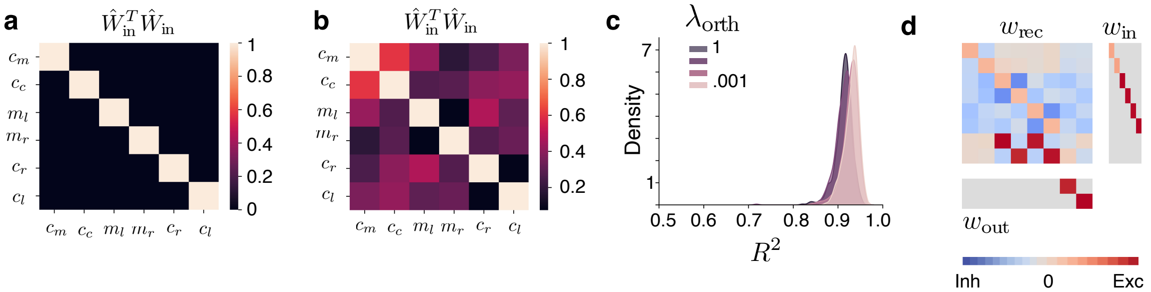

Supplementary Figure 6. Effect of input correlations in RNN on latent circuit inference. In RNNs, orthogonality of input and output vectors is determined by the L_2 norm of the off-diagonal entries of the matrix $B^T B$, where B corresponds to the concatenation of the matrices W_{in} and W_{out}^T along their second dimension, with columns normalized to unit length. The degree of orthogonality is controlled during training by the coefficient λ_{orth} of the L_2 penalty. (**a**) Correlation matrix between input vectors for an RNN trained with $\lambda_{\text{orth}} = 1$ shows that inputs are orthogonal. This same hyperparameter value is used for the models in the main text. (**b**) Correlation matrix between input vectors for an RNN with $\lambda_{\text{orth}} = 0$ shows that some correlations in the inputs arise during training if not constrained. (**c**) Distribution of the latent circuit fit quality for an ensemble of 25 RNNs trained with $\lambda_{\text{orth}} = 0$ to allow for correlations in their input. To determine the effect of these correlations on the latent circuit model, we allowed correlations in the latent circuit model by varying an analogous hyperparameter λ_{orth}. For each RNN and λ_{orth}, we then fit 20 latent circuits. Allowing input correlations in the latent circuit improves fits, but only slightly so. (**d**) Mean latent circuit connectivity for $\lambda_{\text{orth}} = 0.001$ shows that allowing input correlations does not change the underlying inhibitory mechanism.

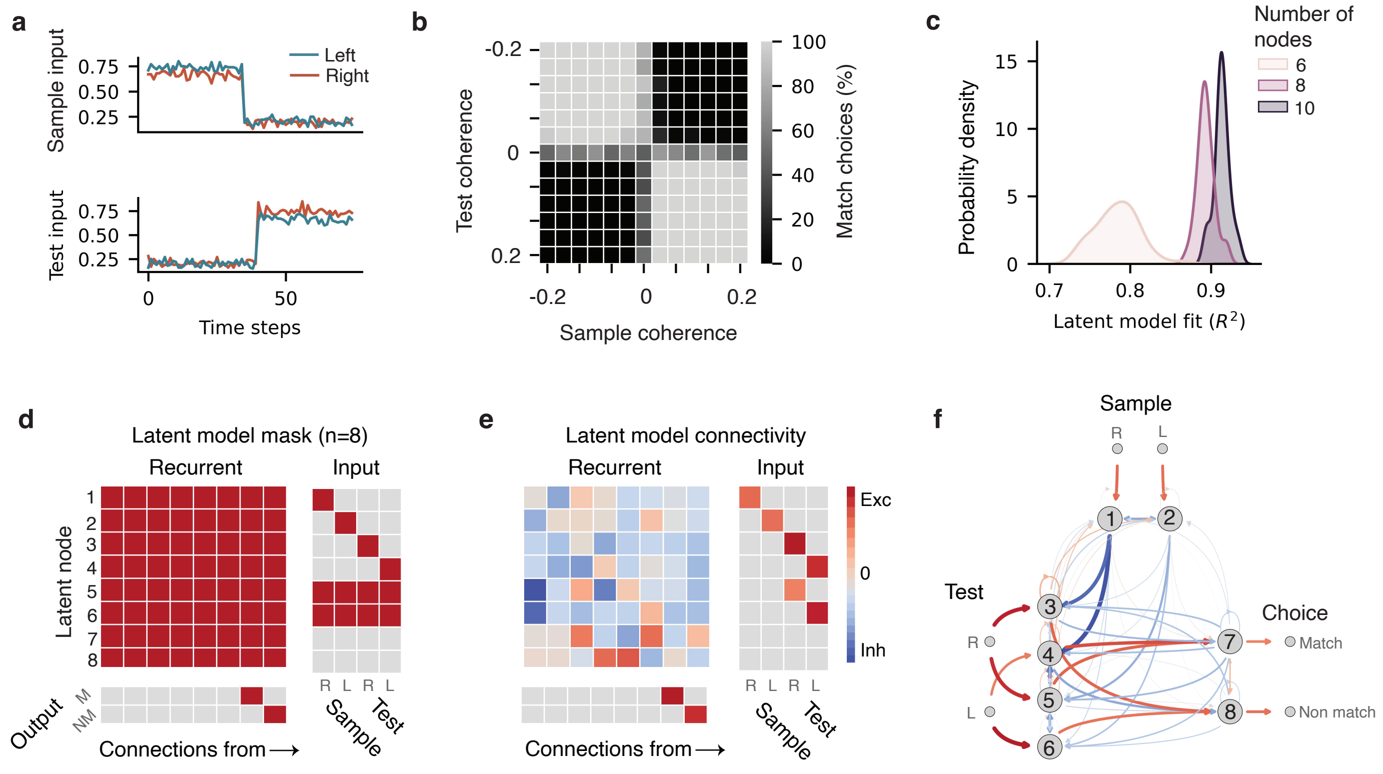

Supplementary Figure 7. Latent circuit mechanism in RNN models of delayed match-to-category task. (**a**) Delayed match-to-category (DMC) task. The model network receives two noisy sample inputs (upper panel) and must categorize them as left or right depending on which input is larger. After a brief delay (5 time steps), the network receives two noisy test inputs and must determine whether the test category (left or right) matches the sample category. (**b**) Psychometric function shows that an example RNN correctly discriminates match and non-match trials. The stimulus coherence determines the difference between the mean strengths of left and right inputs. (**c**) We trained 50 RNNs on the task and fitted responses of each RNN with latent circuit models. The DMC task has 4 inputs (left and right for each sample and test) and 2 outputs (match and non-match). However, latent circuit models with the minimal number of 6 latent nodes (4 input and 2 output nodes) did not provide a good fit (low R^2). The fit quality improved for latent circuits with 8 nodes, and higher number of latent nodes did not significantly increase the fit quality metric. This result is consistent with the dimensionality of the network dynamics: the first eight principal components explained 96% of the total variance. Thus, the DMC task requires at least 8 nodes in the latent circuit model. The distributions show the fit quality metric for 25 latent circuits fitted to each of 50 RNNs, for each number of latent nodes. (**d**) Architecture of the latent circuit model with $n = 8$ nodes. Gray entries in the connectivity matrices are constrained to be zero during training, and red entries are not constrained. The inputs are constrained so that each of the four inputs targets one of the first four nodes, to eliminate redundant solutions corresponding to permutation symmetries among these nodes in the fitted model. The inputs to the two additional latent nodes are not constrained. The last two latent nodes project to the output. (**e**) Connectivity of the fitted latent circuit model for an example RNN reveals an inhibitory mechanism for the DMC task, which is similar to the mechanism for context-dependent decision-making. The model uses two additional latent nodes to create two copies of the test stimulus, each with a different stimulus-to-choice mapping (e.g., input R projects to both nodes 3 and 5, which project to the non-match and match output nodes, respectively). Two nodes receiving sample inputs (1 and 2) inhibit one of these alternative stimulus to choice mappings, thus selecting the match or non-match response pathway. (**f**) Circuit diagram representation of the latent connectivity in e.

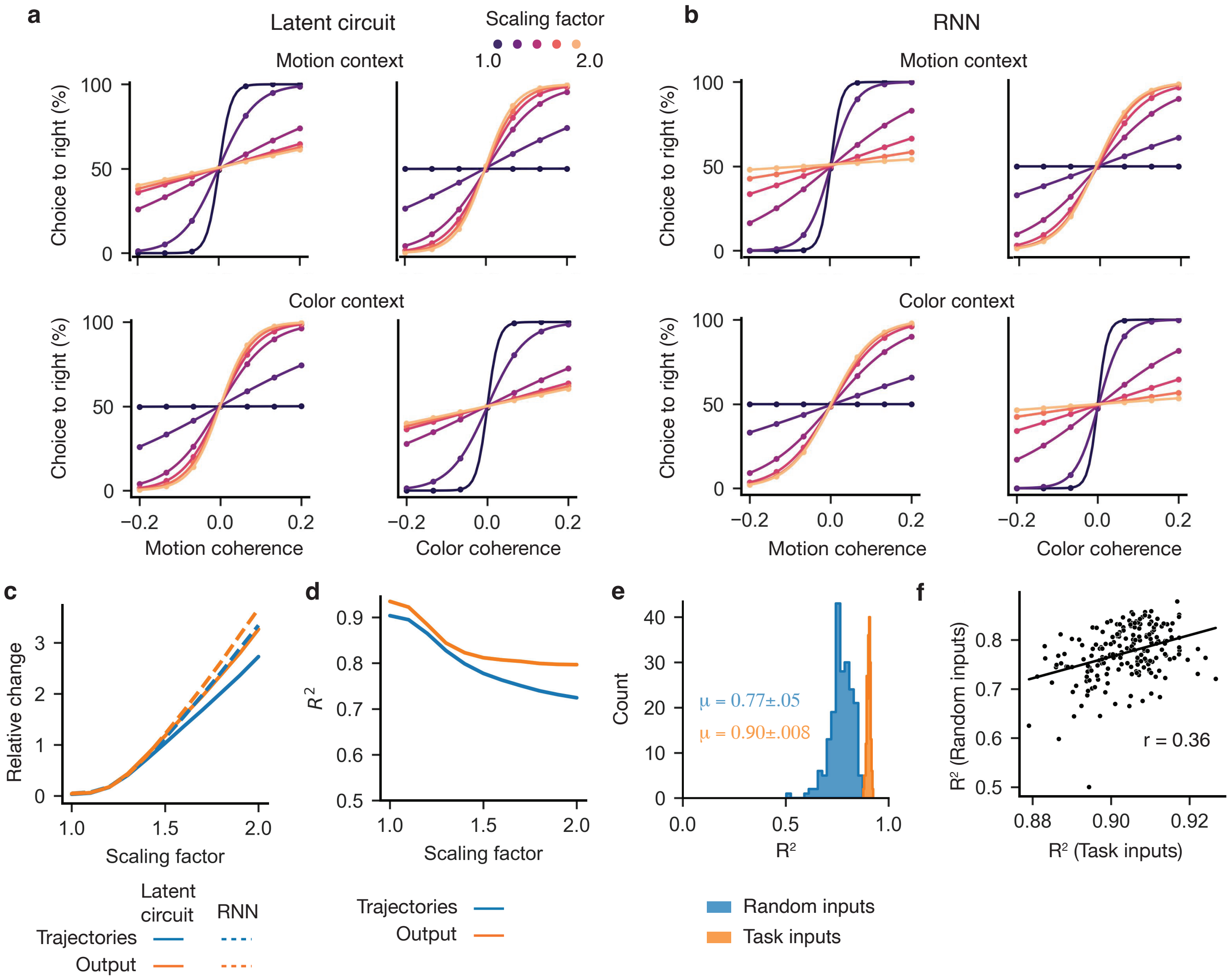

Supplementary Figure 8. Testing latent circuit predictions on out-of-distribution inputs. We tested the ensemble of 200 RNNs and their latent circuits (Fig. 5 in the main text) on two types of out-of-distribution inputs: 1) scaling the amplitude of task inputs (a-d), and 2) random inputs not adhering to the task structure (e,f). (**a**) In each context, we scaled the amplitude of the irrelevant sensory inputs by a factor s ranging from 1 to 2, which had a significant effect on the psychometric function of latent circuits. At $s = 2$, the latent circuits made choices based on the irrelevant stimulus and ignoring the relevant stimulus, which is completely opposite to the normal task behavior. For each scaling factor, the psychometric function is averaged across 200 latent circuit models for the RNN ensemble. (**b**) The average changes in the psychometric function of RNNs were highly consistent with predictions of their latent circuit models (cf. to a), showing that latent circuits accurately predicted changes in task behavior for out-of-distribution inputs. (**c**) We further quantified the corresponding changes in latent trajectories by computing the relative change defined as the mean squared deviation between trajectories for normal and scaled inputs normalized by the total variance in the normal task trajectories. At $s = 2$, the deviation of the latent trajectories from the normal task trajectories was three-fold greater than their total variance. (**d**) The average latent circuit prediction accuracy (R^2) shows only a moderate decrease with the scaling factor for both the latent RNN trajectories (blue) and RNN outputs (orange). The prediction accuracy stays fairly high ($R^2 > 0.7$) even for the three-fold deviation from the normal task trajectories at $s = 2$. (**e**) We generated 2,000 random inputs that were constant in time and had mean and standard deviation matched to the normal task inputs. Distribution of R^2 for latent circuit predictions of RNN trajectories for these random inputs (blue) shows only a moderate decrease in prediction accuracy relative to the normal task inputs (orange). (**f**) The prediction accuracy for random inputs (y-axis) correlated with the quality of the original latent circuit fit (x-axis, correlation coefficient $r = 0.36$), indicating that latent circuits that better fit RNN responses also better generalize to out-of-distribution inputs.
