# Supplementary material for: Latent circuit inference from heterogeneous neural responses during cognitive tasks
Source: Nat Neurosci. 2025 Feb 10;28(3):665–75. doi: 10.1038/s41593-025-01869-7 (PMC11893458; doi:10.1038/s41593-025-01869-7)
Supplement: Supplementary file 2 — Reporting Summary [file 41593_2025_1869_MOESM2_ESM.pdf]

nature portfolio

Corresponding author(s): Tatiana Engel

Last updated by author(s): Nov 1, 2024

Reporting Summary

Nature Portfolio wishes to improve the reproducibility of the work that we publish. This form provides structure for consistency and transparency in reporting. For further information on Nature Portfolio policies, see our Editorial Policies and the Editorial Policy Checklist.

Statistics

For all statistical analyses, confirm that the following items are present in the figure legend, table legend, main text, or Methods section.

n/a	Confirmed	
☐	☒	The exact sample size (*n*) for each experimental group/condition, given as a discrete number and unit of measurement
☐	☒	A statement on whether measurements were taken from distinct samples or whether the same sample was measured repeatedly
☐	☒	The statistical test(s) used AND whether they are one- or two-sided<br>*Only common tests should be described solely by name; describe more complex techniques in the Methods section.*
☐	☒	A description of all covariates tested
☒	☐	A description of any assumptions or corrections, such as tests of normality and adjustment for multiple comparisons
☐	☒	A full description of the statistical parameters including central tendency (e.g. means) or other basic estimates (e.g. regression coefficient) AND variation (e.g. standard deviation) or associated estimates of uncertainty (e.g. confidence intervals)
☐	☒	For null hypothesis testing, the test statistic (e.g. *F*, *t*, *r*) with confidence intervals, effect sizes, degrees of freedom and *P* value noted<br>*Give P values as exact values whenever suitable.*
☒	☐	For Bayesian analysis, information on the choice of priors and Markov chain Monte Carlo settings
☒	☐	For hierarchical and complex designs, identification of the appropriate level for tests and full reporting of outcomes
☐	☒	Estimates of effect sizes (e.g. Cohen's *d*, Pearson's *r*), indicating how they were calculated

Our web collection on statistics for biologists contains articles on many of the points above.

Software and code

Policy information about availability of computer code

Data collection	The neurophysiological data were previously described in Ref. 19 (Mante et al., Nature, 503, 78, 2013). Data were recorded using the MAP data-acquisition system (Plexon Inc., Dallas, TX). Spike sorting was conducted by clustering based on principal component analysis using the Plexon offline sorter (Plexon Inc., Dallas, TX).
Data analysis	Custom Python code was used for data analyses. Plots were generated with Python (version 3.8) and Seaborn (version 0.9.0). Recurrent neural networks were trained using PyTorch (version 2.4). The source code to reproduce results of this study is available on GitHub (https://github.com/engellab/latentcircuit) and Zenodo (https://zenodo.org/records/14020108).

For manuscripts utilizing custom algorithms or software that are central to the research but not yet described in published literature, software must be made available to editors and reviewers. We strongly encourage code deposition in a community repository (e.g. GitHub). See the Nature Portfolio guidelines for submitting code & software for further information.

Data

Policy information about availability of data

All manuscripts must include a data availability statement. This statement should provide the following information, where applicable:

- Accession codes, unique identifiers, or web links for publicly available datasets
- A description of any restrictions on data availability
- For clinical datasets or third party data, please ensure that the statement adheres to our policy

Synthetic data were generated using custom Python 3 code and can be reproduced using the source code with model parameters included in the Source Data files provided with this paper. The neurophysiological data were previously described in Ref. 19 (Mante et al., Nature, 503, 78, 2013) and are available in the public repository https://www.ini.uzh.ch/en/research/groups/mante/data.html for download.

Research involving human participants, their data, or biological material

Policy information about studies with human participants or human data. See also policy information about sex, gender (identity/presentation), and sexual orientation and race, ethnicity and racism.

Reporting on sex and gender	N/A
Reporting on race, ethnicity, or other socially relevant groupings	N/A
Population characteristics	N/A
Recruitment	N/A
Ethics oversight	N/A

Note that full information on the approval of the study protocol must also be provided in the manuscript.

Field-specific reporting

Please select the one below that is the best fit for your research. If you are not sure, read the appropriate sections before making your selection.

☒ Life sciences ☐ Behavioural & social sciences ☐ Ecological, evolutionary & environmental sciences

For a reference copy of the document with all sections, see nature.com/documents/nr-reporting-summary-flat.pdf

Life sciences study design

All studies must disclose on these points even when the disclosure is negative.

Sample size	The neurophysiological data were previously described in Ref. 19 (Mante et al., Nature, 503, 78, 2013). The number of trials in each session was determined by the animals' ability to perform the task. The number of simultaneously recorded neurons was determined by the characteristics of the recording technique. The number of subjects (2) is standard for the primate studies.
Data exclusions	Analysis windows for both monkeys started 100 ms after stimulus onset and continued for 750 ms after stimulus onset. All recorded units that had at least four correct trials in each task condition were included in the analysis.
Replication	Findings were successfully replicated in two macaque monkeys.
Randomization	No randomization was performed. Only one experimental group exists.
Blinding	No blinding was performed. Only one experimental group exists.

Reporting for specific materials, systems and methods

We require information from authors about some types of materials, experimental systems and methods used in many studies. Here, indicate whether each material, system or method listed is relevant to your study. If you are not sure if a list item applies to your research, read the appropriate section before selecting a response.

Materials & experimental systems

n/a	Involved in the study
☒	☐ Antibodies
☒	☐ Eukaryotic cell lines
☒	☐ Palaeontology and archaeology
☐	☒ Animals and other organisms
☒	☐ Clinical data
☒	☐ Dual use research of concern
☒	☐ Plants

Methods

n/a	Involved in the study
☒	☐ ChIP-seq
☒	☐ Flow cytometry
☒	☐ MRI-based neuroimaging

Animals and other research organisms

Policy information about studies involving animals; ARRIVE guidelines recommended for reporting animal research, and Sex and Gender in Research

Laboratory animals	The animals were adult males, ages 13 (monkey A) and 8 (monkey F) years old at the time the experiments were conducted.
Wild animals	The study did not involve wild animals.
Reporting on sex	*Indicate if findings apply to only one sex; describe whether sex was considered in study design, methods used for assigning sex. Provide data disaggregated for sex where this information has been collected in the source data as appropriate; provide overall numbers in this Reporting Summary. Please state if this information has not been collected. Report sex-based analyses where performed, justify reasons for lack of sex-based analysis.*
Field-collected samples	The study did not involve field-collected samples.
Ethics oversight	No ethical approval was necessary as all neurophysiological data were obtained from a public repository. The data were collected and previously described in Ref. 19 (Mante et al., Nature, 503, 78, 2013). All surgical and behavioral procedures conformed to the guidelines established by the National Institutes of Health and were approved by the Institutional Animal Care and Use Committee of Stanford University.

Note that full information on the approval of the study protocol must also be provided in the manuscript.

Plants

Seed stocks	*Report on the source of all seed stocks or other plant material used. If applicable, state the seed stock centre and catalogue number. If plant specimens were collected from the field, describe the collection location, date and sampling procedures.*
Novel plant genotypes	*Describe the methods by which all novel plant genotypes were produced. This includes those generated by transgenic approaches, gene editing, chemical/radiation-based mutagenesis and hybridization. For transgenic lines, describe the transformation method, the number of independent lines analyzed and the generation upon which experiments were performed. For gene-edited lines, describe the editor used, the endogenous sequence targeted for editing, the targeting guide RNA sequence (if applicable) and how the editor was applied.*
Authentication	*Describe any authentication procedures for each seed stock used or novel genotype generated. Describe any experiments used to assess the effect of a mutation and, where applicable, how potential secondary effects (e.g. second site T-DNA insertions, mosiacism, off-target gene editing) were examined.*
